# Targeting *Penicillium expansum* GMC Oxidoreductase with High Affinity Small Molecules for Reducing Patulin Production

**DOI:** 10.3390/biology10010021

**Published:** 2020-12-31

**Authors:** Vincenzo Tragni, Pietro Cotugno, Anna De Grassi, Maria Maddalena Cavalluzzi, Annamaria Mincuzzi, Giovanni Lentini, Simona Marianna Sanzani, Antonio Ippolito, Ciro Leonardo Pierri

**Affiliations:** 1 Department of Soil, Plant and Food Sciences, University of Bari Aldo Moro, Via Amendola 165/A, 70126 Bari, Italy; vincenzo.tragni@uniba.it (V.T.); annamaria.mincuzzi@uniba.it (A.M.); 2 Biology Department, University of Bari Aldo Moro, Via Amendola 165/A, 70126 Bari, Italy; pietro.cotugno@uniba.it; 3 Laboratory of Biochemistry, Molecular and Structural Biology, Department of Biosciences, Biotechnologies, Biopharmaceutics, University of Bari, Via E. Orabona, 4, 70125 Bari, Italy; anna.degrassi@uniba.it; 4 BROWSer S.r.l., c/o Department of Biosciences, Biotechnologies, Biopharmaceutics, University “Aldo Moro” of Bari, Via E. Orabona, 4, 70126 Bari, Italy; 5 Dipartimento di Farmacia—Scienze del Farmaco, Università degli Studi di Bari Aldo Moro, via Orabona 4, 70125 Bari, Italy; mariamaddalena.cavalluzzi@uniba.it (M.M.C.); giovanni.lentini@uniba.it (G.L.); 6 CIHEAM Bari, Via Ceglie 9, 70010 Valenzano (BA), Italy

**Keywords:** *Penicillium expansum* GMC oxidoreductase, patulin biosynthesis, contamination prevention, mycotoxin production inhibition, comparative 3D modeling, virtual screening of chemical libraries

## Abstract

**Simple Summary:**

With the urgent necessity of potential treatments for limiting mycotoxin production and postharvest fungal rots, we propose a combined in silico/in vitro/in vivo strategy for the rapid and effective identification of bioactive small molecules, chosen among a chemical library hosting approved drugs and phytochemicals, to be used after harvest. The molecular target of our analysis was the GMC oxidoreductase from *Penicillium expansum* involved in the biosynthesis of patulin, a mycotoxin that can contaminate many foods, especially fruits and fruit-based products. The employed in silico/in vitro/in vivo assays described in our study proved the effectiveness of our strategy and in particular of two small molecules, 6-hydroxycoumarin (structurally related to umbelliferon, an already characterized patulin synthase inhibitor) and meticrane (an already approved drug) in reducing patulin accumulation. Our findings highly recommend the mentioned ligands to be subjected to further analysis for being used in the next future in place of other more toxic compounds, in postharvest treatments based on dipping or drenching methods.

**Abstract:**

Flavine adenine dinucleotide (FAD) dependent glucose methanol choline oxidoreductase (GMC oxidoreductase) is the terminal key enzyme of the patulin biosynthetic pathway. GMC oxidoreductase catalyzes the oxidative ring closure of (*E*)-ascladiol to patulin. Currently, no protein involved in the patulin biosynthesis in *Penicillium expansum* has been experimentally characterized or solved by X-ray diffraction. Consequently, nothing is known about *P. expansum* GMC oxidoreductase substrate-binding site and mode of action. In the present investigation, a 3D comparative model for *P. expansum* GMC oxidoreductase has been described. Furthermore, a multistep computational approach was used to identify *P. expansum* GMC oxidoreductase residues involved in the FAD binding and in substrate recognition. Notably, the obtained 3D comparative model of *P. expansum* GMC oxidoreductase was used for performing a virtual screening of a chemical/drug library, which allowed to predict new GMC oxidoreductase high affinity ligands to be tested in in vitro/in vivo assays. In vitro assays performed in presence of 6-hydroxycoumarin and meticrane, among the highly affinity predicted binders, confirmed a dose-dependent inhibition (17–81%) of patulin production by 6-hydroxycoumarin (10 µM–1 mM concentration range), whereas the approved drug meticrane inhibited patulin production by 43% already at 10 µM. Furthermore, 6-hydroxycoumarin and meticrane caused a 60 and 41% reduction of patulin production, respectively, in vivo on apples at 100 µg/wound.

## 1. Introduction

Glucose methanol choline (GMC) oxidoreductase superfamily is a large family of proteins specialized in a wide variety of biochemical oxidative reactions. GMC oxidoreductases are mainly divided in seven subgroups, share a common structural fold, host a covalently or non-covalently bound Flavin Adenine Dinucleotide (FAD) cofactor and show a similarly located substrate binding region. Notably, among the characterized GMC oxidoreductases, cellobiose dehydrogenases (CDH) host a *b*-type cytochrome domain [1]. 

Biochemical analysis and sequence comparison of GMC oxidoreductases have revealed strictly conserved Rossmann fold (βαβαβ) [2] or βαβ mononucleotide-binding motif [3] involved in the binding of FAD, while sequence variation reflects the ability of the investigated GMC oxidoreductases in oxidizing selectively one substrate or a class of chemically related ligands.The overall reaction mechanism for these FAD-dependent oxidoreductases is quite well conserved [3].

*Penicillium expansum*, a ubiquitous phytopathogen causing postharvest destructive rots in a variety of commodities, but mainly in apples [4,5], is responsible for the production of the mycotoxin patulin that exerts detrimental effects on mammals’ health [4,5]. A GMC oxidoreductase, plays a key role in patulin biosynthesis. Coded by the gene *patE*, it is located both in *P. expansum* vacuoles or attached to the fungal cell wall. This GMC oxidoreductase appeared to be involved in the last step of patulin biosynthetic pathway, i.e., the conversion of *(E)*-ascladiol (not toxic to human cells [6]), to patulin, when GMC oxidoreductase is secreted through vacuole fusion with the fungal cell wall [7]. Although the GMC oxidoreductase knockout proved to not affect *P. expansum* colony growth and sporulation, as compared to the wild type [8], Tannous and co-workers [6] demonstrated that a *P. expansum* mutant knock out for *patE* did not produce patulin, but accumulated high levels of (*E*)-ascladiol. 

Being patulin detrimental to human and other mammalian health, and reasonably involved in *P. expansum* pathogenicity/virulence [4,5], patulin content in fruits, vegetables and their derivatives has been regulated in several countries, including those of the European Union. Reducing patulin production might also help in limiting economic losses due to the excess of patulin detected in fresh fruits/vegetables and their derived processed products, with specific reference to baby foods [4,5]. 

In light of the existing guidelines, inhibiting *P. expansum* GMC oxidoreductase might represent an efficient strategy for reducing/preventing patulin production by the fungus [4,5]. 

Indeed, since no/few treatments are allowed for dealing with patulin accumulation related problems in the postharvest handling of fruits and vegetables [4,5], there is an increasing need of new strategies alternative to the employment of conventional fungicides. However, the selection of molecules able to reduce mycotoxin production is often time-consuming, expensive and inconclusive.

In this study, the 3D comparative model of *P. expansum* GMC oxidoreductase was built and used for performing a virtual screening of a chemical library, to predict small molecules with high affinity for *P. expansum* GMC oxidoreductase, chosen among a library of approved drugs or phytochemicals. The predicted high affinity small molecules have been tested in in vitro/in vivo assays, aiming to identify chemicals able to control patulin production and/or the rot caused by the fungus.

## 2. Materials and Methods

### 2.1. Sampling of Protein Sequences and Crystallized Structures

The amino acid protein sequence under the code XP_016595360.1 of *P. expansum* GMC oxidoreductase [6,8,9] was retrieved from the National Centre for Biotechnology Information (https://www.ncbi.nlm.nih.gov/protein/). 

Homologous protein sequences of the GMC oxidoreductase superfamily were collected from RefSeq protein database (http://www.ncbi.nlm.nih.gov.refseq/) by using blastp (https://blast.ncbi.nlm.nih.gov/) searches (with default parameters). According to Sützl et al. [3], specific proteins were used as query sequences for sampling the related homologous sequences from other fungi within the refseq protein database, namely: two alcohol oxidases (AOx), one from *Ogataea angusta* (CAA26278.1) and one from *Phanerochaete chrysosporium* (CDG66232.1); two aryl-alcohol oxidases (AAO), one from *Pleurotus pulmonarius* (AAF31169.1) and one from *Pycnoporus cinnabarinus* (ALS87661.1); two cellobiose dehydrogenases (CDH), one from *Myriococcum thermophilum* (syn. *Crassicarpon hotsonii*, ABS45567.2) and one from *Trametes cinnabarina* (ADX41688.1); two glucose dehydrogenases (GDH), one from *Aspergillus flavus* (XP_002372599.1) and one from *Pycnoporus cinnabarinus* (AIL89873.1); two glucose oxidases (GOx), one from *Aspergillus niger* (AGI04246.1) and one from *Talaromyces variabilis* (CAE47418.1); two pyranose dehydrogenases (PDH), one from *Leucoagaricus meleagris* (4H7U.pdb; AAW82997.1) and one from *Agaricus xanthodermus* (AHA85314.1); and two pyranose oxidases (POx), one from *Trametes ochracea* (AAP40332.1) and one from *Tricholoma matsutake* (Q8J2V8.1). Only sequences showing a 30–99% of identical residues with the queries, >90% of coverage and an E-value < 10^−72^ were maintained for the following comparative analyses. Furthermore, in order to study relationships between the sampled GMC oxidoreductases and *P. expansum* GMC oxidoreductase, the latter was used as a query sequence for sampling all the GMC oxidoreductases phylogenetically close to the investigated enzyme. 

### 2.2. Multiple Sequence Alignment (MSA) and Phylogenetic Analyses

ClustalW was used to build a MSA of the sampled sequences and manually optimized by using Jalview (according to protocols previously described [10,11,12,13,14,15]). The analysis of the evolutionary relationships among homologous sequences was performed by using MEGA5 [16] starting from a MSA of 125 protein sequences sampled as described above. Notably, the tree was built from the un-gapped MSA applying the Maximum Likelihood Method with the JTT model [16] for the amino acid substitutions and a gamma distribution (five discrete gamma categories) for the rates among sites. The robustness of the tree was tested by applying a total of 150 bootstrap samplings.

### 2.3. Comparative 3D Modelling

For our comparative analyses the sequences of the available GMC oxidoreductase crystallized structures were also sampled by using fold recognition methods [15,17]. With this aim, the amino acid sequence of *P. expansum* GMC oxidoreductase was used as a query sequence for identifying homologous crystallized structures within the Protein Data Bank by using I-Tasser [18], pGenThreader [19], and in-house developed scripts [17,20].

The 3D comparative model of *P. expansum* GMC oxidoreductase was built starting from the sequence-structure pairwise alignment of *P. expansum* GMC oxidoreductase and the *Pleurotus eryngii* aryl-alcohol oxidase (AAO, “5oc1.pdb” [21]), crystallized in complex with *p*-anisic acid [15,22]. The obtained pairwise alignment was imported in SwissPDBViewer [23,24] and the 3D comparative model of *P. expansum* GMC was built and energetically minimized. Finally, PyMOL [25] (http://www.pymol.org) was used for examining the obtained 3D comparative model to check the correct packing of the secondary structures and verify the presence of putative clashes. 

Notably, considering that all the GMC oxidoreductases host a FAD cofactor in a similarly located binding region [3,26,27], the 3D comparative model of the *P. expansum* GMC oxidoreductase was superimposed to “5oc1.pdb” and FAD cofactor was duplicated from “5oc1.pdb”, and docked in the *P. expansum* GMC oxidoreductase aligned/superimposed binding region. 

Similarly, considering that all the GMC oxidoreductases host a similarly located substrate binding region with two characteristic histidine residues (with the exception of 5ncc.pdb hosting a histidine and an arginine residue [28]), the 3D comparative model of the *P. expansum* GMC oxidoreductase was superimposed to the crystallized GMC oxidoreductases: “5oc1.pdb”, hosting *p*-anisic acid in the catalytic binding pocket, [29]; “4ha6.pdb”, hosting 4-(aminoethyl)-5-(hydroxymethyl)-2-methylpyridin-3-ol in its catalytic binding pocket, [30]; “5ncc.pdb”, hosting palmitic acid in its catalytic binding pocket [28]; “3gdn.pdb” and “6lqy.pdb”, hosting benzaldehyde in their catalytic binding pockets [31,32] for highlighting a protein region that contained all the cited crystallized superimposed ligands. The obtained protein region was supposed to be involved in the binding of patulin and/or its (*E*)-ascladiol precursor and explored for investigating patulin/(*E*)-ascladiol binding modes and new *P. expansum* GMC oxidoreductase high affinity ligands to be tested in in vitro assays.

The PyMOL “super” command was used for superimposing the analysed GMC oxidoreductases. This command returns a structural alignment of the investigated proteins based on a sequence-independent structure-based pairwise alignment. In addition, the “super” command is also able to return a possible superimposition of proteins with a lower sequence similarity [10,11,33,34,35].

### 2.4. Virtual Screening of a Chemical Library

The obtained 3D comparative model of *P. expansum* GMC oxidoreductase was used for performing a virtual screening of a chemical library by using Autodock4.2 [36]. 

Before running the virtual screening, the 3D model was optimized for the docking simulation by converting the GMC oxidoreductase “.pdb” file, obtained by SwissPDBViewer, in a “.pdbqt” file, by computing, Gasteiger charges for each atom of the original GMC oxidoreductase “.pdb” file [37]. 

The gridbox used for the virtual screening contained all the residues within 4 Å from the cited superimposed crystallized ligands and consisted of 54 × 54 × 64 gridpoints in the x–y–z cartesian space, with the gridbox center consisting of coordinates x: −71.315; y: 21.495; z: 11.228. For each virtual screening analysis, the spacing parameter (namely the distance between two adjacent gridpoints within the gridbox) was set to 0.281 Å, “rmstol” was set to 1.5; “ga_pop_size” was set to 150, “ga_num_evals” was set to 2.5 × 10^5^, and “ga_num_generations” was set to 2.7 × 10^4^ as previously described [13,14,38,39,40]. The number of top individuals to survive to the next generation was set to 5 and a ranked cluster analysis of the generated 60 ligand poses was performed. The Lamarckian genetic algorithm was selected for running our analyses.

The screened chemical library consisted of 13215 molecules [38], extracted from the Kegg-compound (drugs and ligands) library and the drug libraries available on https://zinc.docking.org/substances/subsets/endogenous+fda/ and https://www.greenpharma.com/. The library was obtained by selecting all molecules in the 5–2000 g/mol molecular weight range. The ligands of the library were converted in “.mol2” files and energetically optimized (by converting 2D ligands in 3D ligands, and by adding missing hydrogen atoms and charges where necessary) by using OpenBabel (http://openbabel.org/wiki/Main_Page). All the ligands (“.mol2” files) were converted in “.pdbqt” files by using the Autodock MGL tools [37]. Ligands in the result output of the virtual screening were sorted by binding energy.

### 2.5. Quantum Mechanical Calculations

Quantum mechanical calculations were performed for investigating the affinity of the most efficient inhibitors [41]. Briefly, the models of 6-hydroxycoumarin, 7-hydroxycoumarin (umbelliferone), and patulin were built and optimized by the Merck force field MMFF routine [42] implemented in the Spartan’16 suite (Wavefunction Inc., Irvine, CA, USA). 

The structure of patulin was submitted to a systematic conformational analysis with a cut-off of 10 Kcal/mol from the energy of the most stable conformation. 

Only conformers differing by more than 10 degrees in their corresponding solid angles were retained.

Conformers were then classified according to their *ab initio* gas phase energy content calculated at the RHF/3-21G* level. The most stable conformers falling within a window of 5 kcal/mol were retained and submitted to geometry optimization at the RHF/3-21G* level. 

Only conformers differing by more than 5 degrees in their corresponding solid angles were retained and their corresponding energy was calculated at the RHF/6-31G** level.

The conformations falling within the cut-off window (5 kcal/mol) underwent geometry optimization by density functional theory (DFT) with B3LYP functional [43] and the 6-31G* basis set [44]. The so obtained structures were real minima since no imaginary frequency was found in the corresponding calculated IR spectrum (DFT B3LYP/6-31G*//DFT B3LYP/6-31G*). 

### 2.6. Preparation of the Solutions of the Predicted Highly Affinity Ligands

The investigated seven inhibitors as highlighted from the above described virtual screening were purchased from Sigma-Aldrich (Milan, Italy). Stock solutions were freshly prepared, at a concentration of 5 mg/mL by solubilizing saccharin (27.3 mmol/L) and tyrosol (36.2 mmol/L) in sterile distilled water. Meticrane (18.15 mmol/L), *p*-anisic acid (32.86 mmol/L), 6-hydroxycoumarin (32.64 mmol/L), ellagic acid (16.55 mmol/L), and gallic acid (29.4 mmol/L) were prepared by dissolving them into a mixture of phosphate buffer (50.0 mmol/L, pH 7.4) and NaOH (1.0 mol/L, pH 13) (9:1, v/v) as previously reported for 7-hydroxycoumarin (umbelliferone) [45].

### 2.7. Preparation of Patulin Solution

Patulin (purity > 98%) was purchased from Vinci-Biochem SRL (Firenze, Italy) and solubilized into acidified distilled water (pH 4.0 with glacial acetic acid) for preparing a stock solution (5 mg/mL, 32.44 mM), as previously described [5,45]. This stock solution was also used for the HPLC straight-line calibration curve. Five calibrant HPLC solutions were prepared in the range 1–25 µg/mL by diluting the stock standard solution in acidified distilled water [5,45].

### 2.8. Preparation of Penicillium Expansum Conidial Suspension

A toxigenic strain of *P. expansum* (FV4, deposited in the Fungal Collection of the Department of Soil, Plant and Food Sciences of the University of Bari Aldo Moro, Italy) was cultured on Potato Dextrose Agar (PDA, Conda, Madrid, Spain) for 12 days at 24 ± 1 °C in the dark for the production of the inoculum [5,45]. A conidial suspension was prepared by adding 7 mL of a 0.01% Tween 20 (Sigma-Aldrich) solution to the pathogen culture. Afterwards, the fungus was gently scraped to suspend the conidia. The resulting spore suspension was filtered through sterile gauze and the spore counts made by a Thoma counting chamber (HGB Henneberg-Sander GmbH, Lutzellinden, Germany). Sterile distilled water was used for diluting the suspension to the desired concentration [5,45].

### 2.9. In Vitro Assays for Monitoring the Effects of the Predicted GMC Oxidoreductase High Affinity Ligands on Fungal Growth and Patulin Production

In order to quantify *P. expansum* colony growth in presence of the predicted *P. expansum* GMC oxidoreductase high affinity ligands, 1 mL of each investigated compound at four different concentrations (in the range 10 µM–1 mM) was incorporated into melted PDA (at 50 °C). The obtained solutions were poured into the Petri dishes (90 mm Ø, 16 mL per dish). Subsequently, dishes were centrally inoculated in a single point with 10 µL of *P. expansum* conidial suspension (5 × 10^5^ conidia/mL) and incubated at 16 ± 1 °C in the dark for 12 dpi. For each compound at each reported concentration, three replicates of the assay were performed (three agar dishes). The colony diameter was estimated (mm) from the 4th day of incubation at four-day intervals [5,45].

### 2.10. In Vivo Assays for Monitoring the Effects of the Predicted GMC Oxidoreductase High Affinity Ligands on Disease Incidence/Severity and Patulin Production 

#### 2.10.1. Sample Preparation

Freshly harvested Golden Delicious apples (*Malus domestica* L.), free of defects/injuries, and comparable in size, colour and ripeness, were purchased from a fruit market in Bari (Italy). Before treatments, apples were randomized, surface sterilized in a 2% sodium hypochlorite solution for 2 min and rinsed with running tap water for 1 min [5,45].

#### 2.10.2. In Vivo Assays

After air-drying at room temperature for 1 h, apples were wounded by a sterile needle (3 × 3 mm wounds), at 3 equidistant wound-points on the fruit surface and kept at room temperature for 1 h. Then, once dry, a drop (10 µL) of conidial suspension was applied into the same wound. After 12 h, 20 µL of 5 mg/mL stock solution of the investigated compounds, able to reduce patulin production in the previous in vitro assays, were applied into each wound (100 µg/wound). Water/Buffer-treated apples were used as a control. For each treatment, three replicates of 4 apples each were used. Apples of each replicate were put in a plastic tray, packed into a plastic bag for high relative humidity, and incubated at 16 ± 1 °C for 8 d.

Incidence of decay (in terms of percentage of infected wounds) and disease severity (in terms of lesion diameter, mm) were recorded at 2, 4, and 8 days of incubation (dpi), whereas patulin accumulation was determined on treated and control apples by the end of incubation [5,45].

### 2.11. Patulin Analysis

#### 2.11.1. In Vitro Assays

Petri dishes were washed with 3 mL of acidified distilled water for extracting patulin from *P. expansum* mycelium. Washing water was collected, centrifuged at 10,000× *g* for 7 min at room temperature (Beckman centrifuge, Allegra × 22, Fullerton, CA, USA), filtered through a 0.45 µm nylon syringe filter (VWR s.r.l., Milan, Italy) and analysed by HPLC. Results were reported as ppm of patulin per mL of dish washing water, according to our protocols [5,45].

#### 2.11.2. In Vivo Assays

Apple tissue cylinders (9 width × 20 depth mm) were withdrawn by a cork borer from the wounds of each replicate, pooled together, homogenized with a Sorvall Omnimixer (Sorvall Instruments, Norwalk, CT, USA), as according to what previously reported [45]. In summary, pectinase enzyme was used for digesting enzymatically an aliquot (10 g) of each homogenized tissue sample (10 drops with an activity of 1350–1650 U/g) (Orsell S.R.L.) in presence of 10 mL of water and kept overnight at room temperature. Then, the digested samples were centrifuged at 4200× *g* for 15 min, filtered by a filter paper under vacuum (Whatman International Ltd., Middlesex, UK) followed by a 0.45 µm nylon syringe filter (VWR S.R.L), and analysed by HPLC, according to what previously reported [5,45]. Results were reported as µg of patulin per g of fresh apple weight.

#### 2.11.3. Analytical Conditions

Patulin was quantified by injecting 50 µL of the filtrate extract into a liquid chromatographer (Shimadzu corporation, Kyoto, Japan) equipped with a binary gradient pump capable of delivering 1 mL/min constant flow rate (Nexera X2 series gradient pump LC-30AD), a vacuum membrane degasser (DGU-20A3R 3 channel), an autosampler injection system with a 50 µL loop (SIL-30A/C), a column oven set at 30 °C, diode array detector (DAD, SPD-M30A) set at 276 nm, and a chromatography data system for Windows 7 (Data analysis), a Luna (Phenomenex) C18 TMS endcapping column (25 cm l., 4.6 mm i.d., 5.0 μm particle size) (Phenomenex, Torrance, CA, USA). The best conditions for the analyte separation were obtained by using isocratic elution. The mobile phase consisted of acetic acid water solution (0.35 M)/acetonitrile (92/8 v/v). The quantifications were performed by measuring the area of the chromatographic peaks, as previously described [5,45].

### 2.12. Data Statistical Analysis

Statistical analyses were performed by using R (RStudio: Integrated Development for R. RStudio, Inc., Boston, MA, USA; http://www.rstudio.com/) as previously reported [5]. More in detail, at least three independent replicates per sample were performed. Confidence levels and *p*-value thresholds were set to 0.95 and 0.05, respectively, for each test. The Shapiro-Wilk test and the Barlett’s test were performed to preliminarily test the null hypotheses that samples came from populations that are normally distributed and with equal variance, respectively. As the two criteria were satisfied, both for in vitro and in vivo patulin production and radial growth, the parametric ANOVA test and the post hoc Tukey’s test were conducted for quantifying differences among sample groups that underwent different treatments. In the other analyses, i.e., for disease incidence and severity estimation, the Duncan’s Multiple Range Test (DMRT) was used. Percentage data of disease incidence were subjected to arcsine-square-root transformation before the analyses. 

## 3. Results

### 3.1. Evolutionary Relationships among the Sampled GMC Oxidoreductase Homologous Sequences

**Figure 1:**
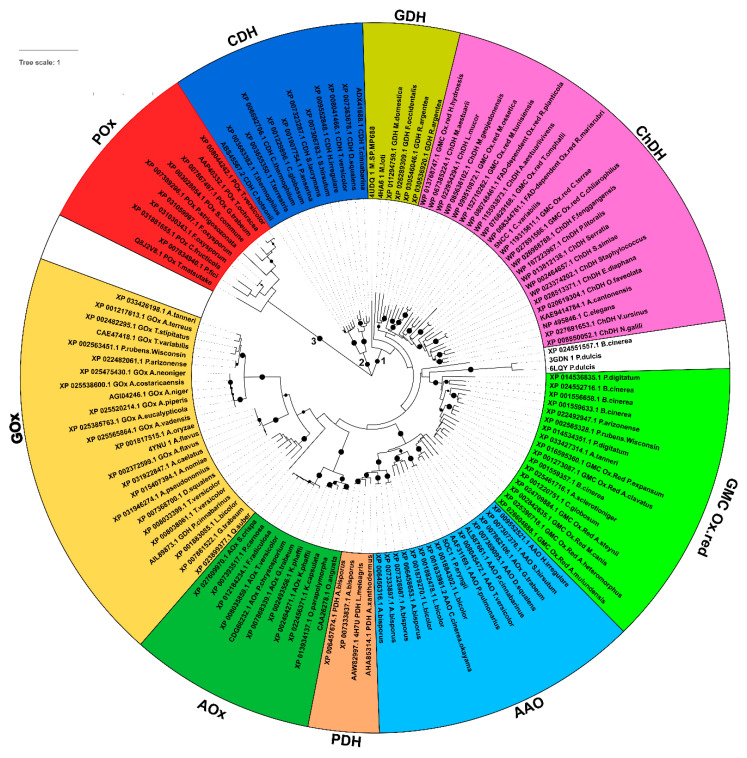
Phylogenetic tree of the sampled GMC oxidoreductase sequences. Maximum likelihood phylogenetic tree of GMC oxidoreductase protein sequences selected from representative taxonomic groups of *Fungi* (with specific attention to *Botrytis, Aspergillus*, and *Penicillium* species among the others), *Plants*, *Metazoa,* and *Bacteria*. Sequence names are reported in correspondence of each leaf, according to the assigned names retrievable together with fasta sequences along blastp searches. Bootstrap values greater than 80% are reported in correspondence of the interested nodes as black circles. The three main subgroups reported in the tree are indicated by numbers “1–3”. Cluster of sequences are grouped and colored based on the sequence relationships highlighted by the phylogenetic analyses. The three sequences on the white background, two sequences about lyases (crystallized in complex with FAD) and one *B. cinerea* sequence are sequence/structurally related to the sampled fungal *P. expansum* related GMC Ox.red sequences, although phylogenetically distant from the other analysed sequences. Abbreviations: AOx, alcohol oxidases; AAO, aryl-alcohol oxidases; CDH, cellobiose dehydrogenases; GDH, glucose dehydrogenases, GOx, glucose oxidases; PDH, pyranose dehydrogenases; POx, pyranose oxidases; GMC Ox.red, *P. expansum* related GMC oxidoreductases; ChDH, choline dehydrogenase.

It has recently been proposed that *P. expansum* patulin synthase, coded by *patE* gene, has a function similar to GMC oxidoreductases [46]. Since it is commonly accepted that all GMC oxidoreductase sequences might share a common ancestral sequence, based on their conserved similar function and overall structure [3,29], we searched for proteins sharing sequence similarity with *P. expansum* GMC oxidoreductase in representative genera from *Fungi* (with specific reference to *Botrytis*, *Aspergillus*, and *Penicillium*), *Plants*, *Metazoa*, and *Bacteria* taxonomic groups and we retrieved 128 putative GMC oxidoreductase homologous proteins. The evolutionary relationships among all these proteins allowed their clustering in three main subgroups further divided into specialized clusters and subclusters, reflecting functional-phylogenetical related specializations (Figure 1). The first subgroup (with a bootstrap value greater than 80%) contains *P. expansum* GMC oxidoreductase homologous sequences together with sequences phylogenetically related to PDH, i.e., see *L. meleagris* AAW82997.1 [47], AAO, i.e., see *P. pulmonarius* AAF31169.1 and *P. cinnabarinus* ALS87661.1 [48], and AOx, i.e., see *P. chrysosporium* CDG66232.1 [49], and GOx, i.e., see *T. variabilis* CAE47418 [50].

This subgroup hosts also the phylogenetically more distant sequences related to ChDH, i.e., see *Staphylococcus* WP_023374202.1 [51], and GDH, i.e., see *P. cinnabarinus* AIL89873.1 [52,53].

The second and third subgroups (both showing bootstrap values greater than 80%) hosts sequences phylogenetically related to CDH, i.e., see *T. cinnabarina* ADX41688.1 [54] POx, i.e., see *T. matsutake* Q8J2V8.1 [55], respectively.

### 3.2. Features of the Sampled GMC Oxidoreductase Homologous Protein Sequences

It was expected that the different eight GMC oxidoreductase subfamilies would have reflected the specialization in species-specific biochemical pathways based on the existence of further well-conserved specific sequence features detectable within a MSA hosting all the sampled sequences, despite of the 30% to 99% identical residues shared by the sampled full-length sequences.

**Figure 2:**
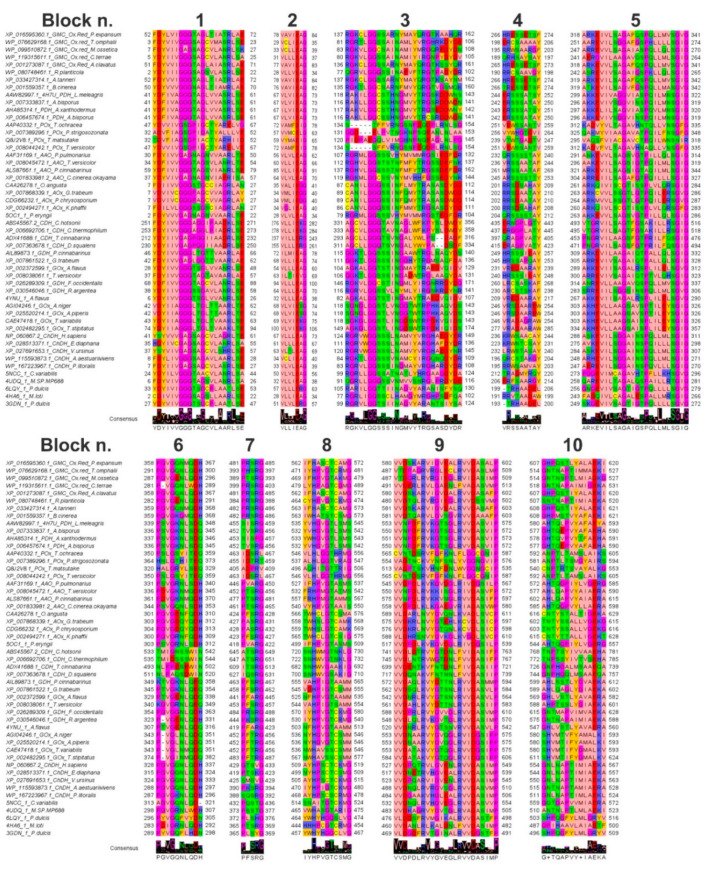
GMC oxidoreductase conserved amino acid blocks. Blocks of conserved amino acids detected by comparing the GMC oxidoreductases sampled through blastp searches and aligned by ClustalW. The highlighted amino acid blocks are numbered (1–10, following their description reported in Section 3.2) and revealed crucial protein regions involved in the FAD binding and/or in the binding of the substrate or more in general residues important for the protein function mechanism. The jalview Zappo color style was used for coloring amino acids. Logo representation is also reported to highlight the most conserved residues.

Indeed, by inspecting the MSA of GMC oxidoreductase sampled homologous proteins, it was possible to highlight 10 amino acid conserved blocks. Some of those blocks, i.e., the three glycine-rich regions (respectively the first, third and fifth sequence blocks grouped in Figure 2) were conserved in all the aligned sequences (with few exceptions), underlining a putative common involvement in the protein function/mechanism. 

The other amino acid blocks showed a high degree of amino acid conservation within specific group of residues more conserved in specific taxonomic groups, reflecting a species-specific acquired function or substrate/cofactor specificity/affinity (Figure 2).

As an example, the three glycine-rich motifs were at the level of residues 52-FDYLVIGGGTAGLTIATRL-70, first amino acid block, 137-RGKCLGGSSARNYMAYQRGTKAAHQR-162, third amino acid block) and 318-IVLSAGAFQSPQLLMVSGVG-341, fifth amino acid block, (XP_016595360.1 *P. expansum* GMC oxidoreductase numbering), and aligned with residues 41-YDFIVAGGGTAGLVVASRLSE-61, 118-RAKILGGCSTHNGMVYTRGSKDDWNS-143, and 299-AKKEVIVAGGVIASPQILMNSGIG-322 (AAW82997.1 *L. meleagris* PDH numbering) in PDH homologs; 32-TDVFIAGSGPIACTYARHIID-52, 120-PIDPAEGQLVIMGHNPNQEAGLNLPG-145, and 295-VAQSFVIACGAVCTPQILWNSNIR-318 (Q8J2V8.1 *T. matsutake* POx numbering) in POx homologs; 30-FDYIVVGAGNAGNVVAARLTE-50, 107-RGRMLGGSSSVHYMVMMRGSIEDFDR-132, and 291-ANKEVVLSAGSVGTPILLQLSGIG-314 (AFF31169.1 *P. pulmonarius* AAO numbering) in AAO homologs; 7-VDVIVCGGGPAGCVVAGRLAY-27, 89-CANILGGGSSINFQMYTRASASDWDD-114, and 267-ARKMVVLSSGTLGTPQILERSGVG-287 (CDG66232.1 *P. chrysosporium* AOx numbering) in AOx homologs; 212-YDYIIVGAGPGGIIAADRLSE-232, 293-AGCLLGGGTSVNGALYWLPS--DADF-316, and 453-PNGRVILAAGSFGTPRILFQSGIG-476 (ADX41688.1 *T. cinnabarina* CDH numbering) in CDH homologs; 46-YDYIVVGGGLTGTTVAARLAE-66, 115-GGKTLGGSSSINGAAWTRGLNAQYDS-140, and 309-ARKEVILAAGAIQTPALLQLSGIG-332 (AIL89873.1 *P. cinnabarinus* GDH numbering) in GDH homologs; 43-YDYIIAGGGLTGLTVAAKLSE-63, 118-SGKGLGGSTLINGDSWTRPDKVQIDS-143, and 303-AKHEVLLAAGSAISPLILEYSGIG-326 (CAE47418.1 *T. variabilis* GOx numbering) in GOx homologs; 7-FDYIIVGAGSAGCVLASRLSE-27, 83-RGRVLGGSSSINGMAYVRGHARDYDR-108, and 247-AEREVILSGGAINSPQVLLLSGIG-270 (WP_167223967.1 *P. litoralis* ChDH numbering) in ChDH homologs.

The first highlighted amino acid block hosted an “aromatic-acidic-aromatic” tripeptide at the beginning of the amino acid block and a “basic-acidic” amino acid pair at the end of the same block in most of the sampled sequences, whereas the third and fifth amino acid blocks were enriched in basic and glycine residues. Notably, the fifth amino acid block shows also a further proline residue conserved in most of the aligned sequences (Figure 2).

The second and the ninth highlighted amino acid blocks, located at residues 78-VAVIEAG-84 and 580-VVDSKARVIGVEALRVVDASALP-602 (XP_016595360.1 *P. expansum* GMC oxidoreductase numbering) showed a further well conserved [V/C/I/L/M][V/L/T/A/G/I][D/N/E] tripeptide (see residues aligned with the underlined residues in the motive reported above), repeated two times in the tenth amino acid block (Figure 2). 

This [V/C/I/L/M][V/L/T/A/G/I][D/E] tripeptide appeared to be conserved in all the analysed subfamilies with few exceptions. Indeed, the blocks 78-VAVIEAG-84 and 580-VVDSKARVIGVEALRVVDASALP-602 (XP_016595360.1 *P. expansum* GMC oxidoreductase numbering) aligned with the amino acid blocks 67-VLVIEAG-73 and 553-VVNPDFKVKGTSGLRVVDASVIP-575 (AAW82997.1 *L. meleagris* PDH numbering) in PDH homologs; 59-VYMAEIG-65 and 513-VADPTSKVHNFDNLWVGGNGCIP-535 (Q8J2V8.1 *T. matsutake* POx numbering) in POx homologs; 56-VLVLEAG-62 and 545-VVDPDLKVKGVDGLRIVDGSILP-567 (AFF31169.1 *P. pulmonarius* AAO numbering) in AAO homologs; 34-VMLIEGG-40 and 576-VVDKRLNVYGTQNLKCVDLSICP-598 (CDG66232.1 *P. chrysosporium* AOx numbering) in AOx homologs; 237-VILLERG-243 and 699-VVDENTKVFNTDNLFIVDASIIP-721 (ADX41688.1 *T. cinnabarina* CDH numbering) in CDH homologs; 72-ILMIEAG-78 and 572-VVDAQLKVYDTTNLRVVDASMMP-594 (AIL89873.1 *P. cinnabarinus* GDH numbering) in GDH homologs; 69-VLVIEKG-75 and 553-VVDATAKVYGTQGLRVVDGSIPP-575 (CAE47418.1 *T. variabilis* GOx numbering) in GOx homologs, 33-VLVLEAG-39 and 484-VVDGAAKVHGLEALRVVDASIMP-506 (WP_167223967.1 *P. litoralis* ChDH numbering) in ChDH homologs (Figure 2).

There was a typical RG highly conserved dipeptide in the third and seventh conserved amino acid blocks consisting of residues previously cited for the third amino acid block and residues 481-PRSRG-485 for the seventh amino acid block (XP_016595360.1 *P. expansum* GMC oxidoreductase numbering) aligning with the amino acid blocks 455-SISRG-459 (AAW82997.1 *L. meleagris* PDH numbering) in PDH homologs; 405-ADPRV-409 (Q8J2V8.1 *T. matsutake* POx numbering) in POx homologs; 466-PVARG-450 (AFF31169.1 *P. pulmonarius* AAO numbering) in AAO homologs; 428-PASRG-432 (CDG66232.1 *P. chrysosporium* AOx numbering) in AOx homologs; 609-ITSRG-613 (ADX41688.1 *T. cinnabarina* CDH numbering) in CDH homologs; 463-PFSRG-467 (AIL89873.1 *P. cinnabarinus* GDH numbering) in GDH homologs; 452-PFTRG-456 (CAE47418.1 *T. variabilis* GOx numbering) in GOx homologs, 388-SQSRG-392 (WP_167223967.1 *P. litoralis* ChDH numbering) in ChDH homologs (Figure 2).

The eighth and tenth amino acid blocks hosted the two crucial histidine residues involved in substrate binding. Notably, the first histidine appeared to be conserved in all the investigated GMC oxidoreductase sampled homologs, whereas the second histidine was replaced by an asparagine in half of the sampled aligned sequences. On this concern it was possible to see the aligned amino acid blocks 562-IFHASCTCAMG-572 and 607-GHPQSTLYALAEKI-620 (XP_016595360.1 *P. expansum* GMC oxidoreductase numbering) in GMC homologs aligned with 535-YVHGVGTLSMS-545 and 580-AHTQLPVYAFAEYA-593 (AAW82997.1 *L. meleagris* PDH numbering) in PDH homologs; 498-HITGTTRI-505 and 540-CNPTRTSVAYALKG-553 (Q8J2V8.1 *T. matsutake* POx numbering) in POx homologs; 527-IFHPVGTASMS-537 and 572-AHTQGPIYLVGERG-585 (AFF31169.1 *P. pulmonarius* AAO numbering) in AAO homologs; 559-TWHSLGTCAMK-569 and 603-TNTYSSALLVGEKG-616 (CDG66232.1 *P. chrysosporium* AOx numbering) in AOx homologs; 682-SNHWVGSAKIG-692 and 726-GNPHGALMSAAEQA-739 (ADX41688.1 *T. cinnabarina* CDH numbering) in CDH homologs; 555-VAHPIGTAAMM-565 and 599-AHLSSTLYGVAEKA-612 (AIL89873.1 *P. cinnabarinus* GDH numbering) in GDH homologs; 536-NWHAVSSCSMM-545 and 580-SHVMTIFYGMALKV-593 (CAE47418.1 *T. variabilis* GOx numbering) in GOx homologs, 468-AYHPSCSCRMG-478 and 511-GNLNAPTIMLAEKL-524 in (WP_167223967.1 *P. litoralis* ChDH numbering) ChDH homologs (Figure 2).

A fourth conserved amino acid block started in most of the aligned sequences with a basic residue (R or H), then in this motif it is possible to observe an alternation of conserved acidic/hydrophilic residue in the middle and an alternation of aromatic/hydrophobic residues at the end of the motif. i.e., you can see residues 266-HRESSETSF-274 (XP_016595360.1 *P. expansum* GMC oxidoreductase numbering) aligning with the amino acid blocks 244-ERSSSATSY-252 (AAW82997.1 *L. meleagris* PDH numbering) in PDH homologs; 239-VTWTGADTV-247 (Q8J2V8.1 *T. matsutake* POx numbering) in POx homologs; 232-QRSSSSTAY-240 (AFF31169.1 *P. pulmonarius* AAO numbering) in AAO homologs; 202-RRSDAATAY-210 (CDG66232.1 *P. chrysosporium* AOx numbering) in AOx homologs; 397-QRAGPVATY-405 (ADX41688.1 *T. cinnabarina* CDH numbering) in CDH homologs; 249- HRSSSIEAY-257 (AIL89873.1 *P. cinnabarinus* GDH numbering) in GDH homologs; 246-VRADAGRAW-254 (CAE47418.1 *T. variabilis* GOx numbering) in GOx homologs; 195-RRWSAMVAY-203 (WP_167223967.1 *P. litoralis* ChDH numbering) in ChDH homologs (Figure 2).

The last conserved amino acid block (the sixth one) was enriched in proline and glycine residue. Notably, at the end of this block an acidic residue was shown in most of the sampled sequences adjacent to an H/N/Q residue, i.e., see the sixth amino acid block at the level of residues 358-PGVGQNMQDH-367 (XP_016595360.1 *P. expansum* GMC oxidoreductase numbering) in GMC homologs aligned with 339-PSVGKNLSDQ-348 (AAW82997.1 *L. meleagris* PDH numbering) in PDH homologs; 320-HALGRYLSEQ-329 (Q8J2V8.1 *T. matsutake* POx numbering) in POx homologs; 331-PSVGRNLSDH-340 (AFF31169.1 *P. pulmonarius* AAO numbering) in AAO homologs; 304-PGVGEQYQDH-313 (CDG66232.1 *P. chrysosporium* AOx numbering) in AOx homologs; 493-NLPPESEWIN-502 (ADX41688.1 *T. cinnabarina* CDH numbering) in CDH homologs; 349-KTVGKNLQEQ-502 (AIL89873.1 *P. cinnabarinus* GDH numbering) in GDH homologs; 343-P-VGLNMQDQ-351(CAE47418.1 *T. variabilis* GOx numbering) in GOx homologs, 287-PGVGKNLQDH-296 in (WP_167223967.1 *P. litoralis* ChDH numbering) ChDH homologs (Figure 2). 

### 3.3. Comparative 3D Modelling of P. expansum GMC Oxidoreductase

pGenThreader and I-TASSER indicated that the crystallized structures of *P. eryngii* aryl-alcohol oxidase “5oc1.pdb”, crystallized in complex with *p*-anisic acid [29], “5ncc.pdb”, crystallized in complex with palmitic acid [28], “4ynu.pdb”, crystallized in complex with d-glucono-1,5-lactone [56], and “4udq.pdb” [57], might be good templates for building the comparative model of *P. expansum* GMC oxidoreductase. The *P. eryngii* aryl-alcohol oxidase “5oc1.pdb” [29] showed the higher percentage of identical residues with *P. expansum* GMC oxidoreductase. Although the percentage of identical residues between *P. expansum* GMC oxidoreductase and the crystallized *P. eryngii* aryl-alcohol oxidase (AAO, “5oc1.pdb”) was only 30%, it was possible to build a reliable sequence-structure pairwise alignment by using residues conserved in the amino acid blocks reported in Figure 2 as anchor points for the pairwise alignment. 

**Figure 3:**
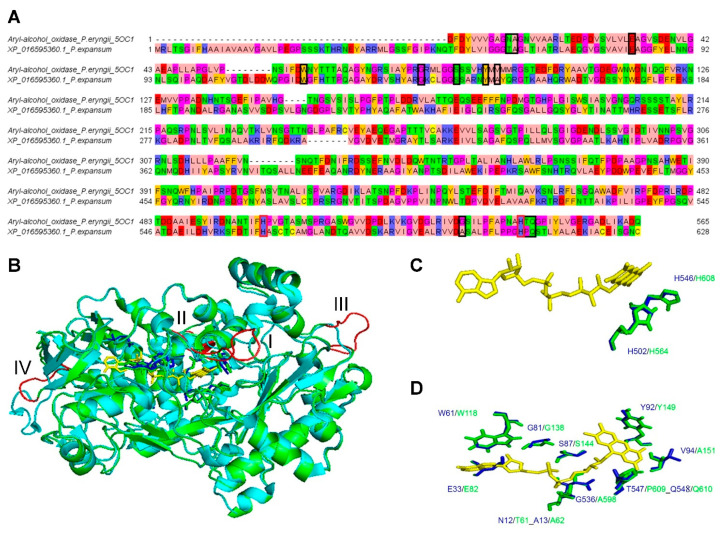
Sequence-structure alignment of *P. expansum* GMC oxidoreductase superimposed to *P. eryngii* aryl-alcohol oxidase. Panel **A**: Black boxes indicate the aligned amino acids involved in the FAD ligand binding, whereas red boxes indicate the aligned amino acids involved in the substrate catalytic binding site. Panel **B**: 3D comparative model of *P. expansum* GMC oxidorectase built by using *P. eryngii* AAO as a protein template. *P. expansum* GMC oxidoreducate is reported in green cartoon representation whereas the AAO crystallized structure from *P. eryngii* is reported in cyan cartoon representation. Loops that show a different orientation between the 3D comparative model and the crystallized superimposed structure are reported in red cartoon. FAD crystallized in AAO superimposed to the *P. expansum* GMC oxidoreductase is reported in yellows sticks. Residues within 4 Å from FAD within AAO and the *P. expansum* GMC oxidoreductase are reported in blue and green sticks, respectively. Panel **C**: zoomed view of the superimposed AAO and the *P. expansum* GMC oxidoreductase histidine residues (within 4 Å from FAD) proposed to be involved in the binding of GMC oxidoreductase substrates. Panel **D**: zoomed view of the superimposed AAO and the *P. expansum* GMC oxidoreductase residues within 4 Å from FAD (His residues are excluded in this panel for a simplified view).

The superimposition of the obtained energetically minimized 3D comparative model with the AAO crystallized structure resulted in an RMSD of 0.257 Å. The main variation between the 3D comparative models and the crystallized structure was observed in correspondence of four loops (loop I, residues, 105-VGTDLDDW-112; loop II, residues, 205-SVLGSG-211; loop III, residues: 295-IRFQDKRA-302, and loop IV, residues:377-NVITQSALLNEE-387; *P. expansum* GMC oxidoreductase residues numbering) (Figure 3). Two of the cited loops (loop I and loop II) kept together helices involved in the packing of local structural elements contributing to the binding of FAD and/or hosting histidine residues and/or other aromatic residues (Y149, amino acid block 3; F563, H564, amino acid block 8; H608, amino acid block 10, *P. expansum* GMC oxidoreductase residues numbering) involved in substrate/cofactor recognition and binding. Notably, the residues involved in the binding of FAD, mainly located among residues of blocks of conserved amino acids 1, 3, 8, and 10, appeared to be conserved both in the MSA (Figure 2) and in the superimposed structures (Figure 3). 

### 3.4. Virtual Screening

Due to the presence of the described conserved amino acid blocks in all the investigated GMC oxidoreductases and assuming that all the investigated GMC oxidoreductase homologous proteins shared a similarly located common substrate binding region, we performed the virtual screening of our chemical library in the gridbox containing residues involved in the binding of all the crystallized ligands available in the homologous crystallized structures, sampled by using the cited folding recognition methods (Table S1).

The list sorted by energy of the 13125 successfully screened ligands, available upon request, included 76 selected for the following structural analyses according to the lowest calculated binding energy and/or to specific structural features. 

More in detail we selected the first 10 small molecules (Table S2) among the 20 best hits because they showed the highest affinities (lowest binding energy) for the investigated binding region and were enriched in aromatic/phenolic functional groups that might fit well *P. expansum* GMC oxidoreductase binding region. Then we chose 11 ligands energetically related to saccharine, structurally related to patulin and (*E*)-ascladiol (Table S3); 11 ligands energetically related to the 7-hydroxycoumarin (umbelliferone) molecule (Table S4), which is known for being able to inhibit patulin production [45]; 11 ligands energetically/structurally related to the *p*-anisic acid (Table S5), known for being able to inhibit “5oc1.pdb” used as a protein template for comparative modelling of *P. expansum* GMC oxidoreductase; 11 ligands structurally/energetically related to the ellagic acid coming from the oxidation of gallic acid and similar in the distribution of the aromatic rings to umbelliferone and other coumarin structurally related small molecules (Table S6), revealed to be able to inhibit patulin production [45]; 11 ligands energetically related to tyrosol, energetically/structurally related to *p*-anisic acid (Table S7), known for being able to inhibit “5oc1.pdb”, and benzaldehyde, known for being a substrate for 3gdn.pdb and 6lqy.pdb, which were among the best protein templates to be used for the comparative modelling of *P. expansum* GMC oxidoreductase. Finally, we selected 11 ligands energetically related to gallic acid (Table S8). Notably, gallic acid is the precursor of ellagic acid.

**Figure 4:**
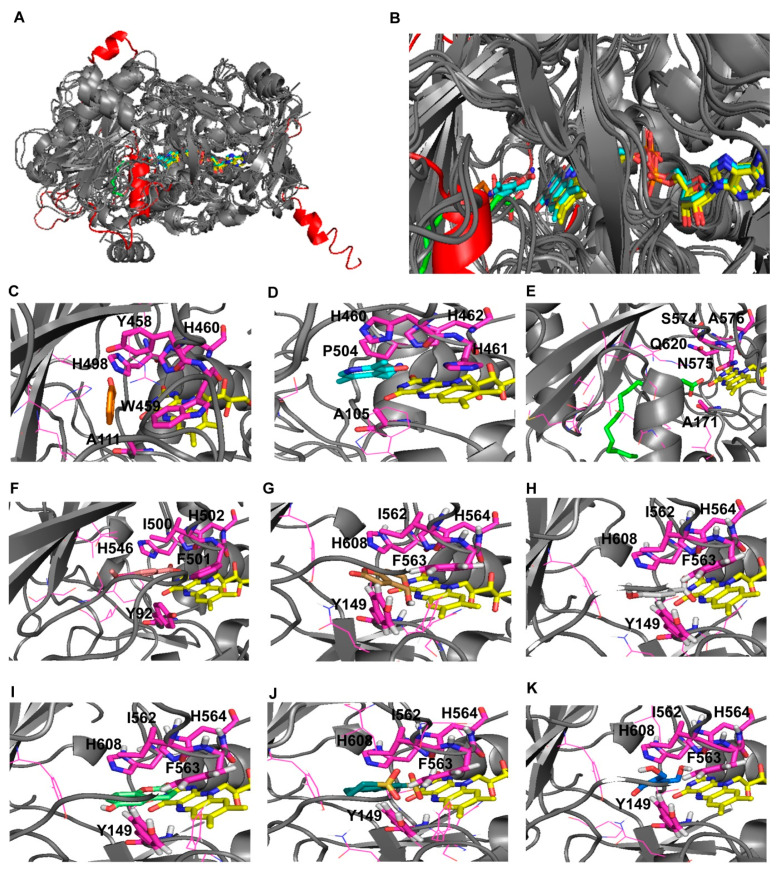
Comparative analysis for identifying the best grid box for Virtual Screening analysis. *P. expansum* GMC comparative model is reported in grey cartoon. FAD is reported in yellow sticks. Panel **A**: superimposition of *P. expansum* GMC oxidoreductase 3D model and crystallized homologous proteins substrates, inhibitors and FAD cofactor. Panel **B**: exploded view of the FAD binding region. Panel **C**: benzaldehyde (HBX) reported in orange sticks representation crystallized within “6lqy.pdb”. Panel **D**: 4-(aminomethyl)-5-(hydroxymethyl)-2-methylpyridin-3-ol (PMX) reported in cyan sticks representation, crystallized within “4ha6.pdb”. panel **E** palmitic acid (PLM) reported in green sticks representation, crystallized within “5ncc.pdb”. Panel **F**: *p*-anisic acid (ANN) reported in light pink sticks representation, crystallized within “5oc1.pdb”. patulin reported in light brown sticks representation (panel **G**); *p*-anisic acid reported in light gray sticks representation (panel **H**); 6-hydroxycoumarin reported in light green sticks representation (panel **I**); meticrane reported in green cyan sticks representation (panel **J**); (*E*)-ascladiol reported in blue sticks representation (panel **K**) within the *P. expansum* GMC oxidoreductase as obtained from docking analyses. Residues numbering of “6lqy.pdb”, shown in panel **C**, and residues numbering of the “6lqy” fasta sequence, shown in Figure 2, differs of 6 units (i.e., A111; Y458, W459, H460 and H498 reported in panel **D** corresponds to A117; Y464, W465, H466 and H504 reported in Figure 2, in blocks 3, 8 and 10, respectively). Residues numbering of “4ha6.pdb”, shown in panel **D**, and residues numbering of the “4ha6” fasta sequence, shown in Figure 2, differs of 16 units (i.e., A105; H460, H461, H462 and P504 reported in panel **D** corresponds to A89; H444, H445, H446 and P488 reported in Figure 2, in blocks 3, 8 and 10, respectively). Residues numbering of “5ncc.pdb” chain A, shown in panel **E**, and residues numbering of the “5ncc” fasta sequence, shown in Figure 2, differs of 60 units (i.e., A171; N575 and Q620 reported in panel **E** corresponds to A111; N515 and Q560 reported in Figure 2, in blocks 3, 8 and 10, respectively). Residues numbering of “5oc1.pdb”, shown in panel **F**, and residues numbering of the “5oc1” fasta sequence, shown in Figure 2, differs of 1 unit (i.e., Y92; I500, F501, H502 and H546 reported in panel **D** corresponds to Y91; I499, F500, H501 and H545 reported in Figure 2, in blocks 3, 8 and 10, respectively).

Among the highlighted 76 ligands we selected seven molecules to be tested in the following in vitro/in vivo assays based on their similarity with patulin, (*E*)-ascladiol and other ligands crystallized in complex with GMC oxidoreductase homologous proteins (Figure 4). All the selected 7 molecules were enriched in aromatic/phenolic functional moieties and two out of the seven molecules show a sulfonamide functional group or a sulfonamide derivative (Table 1).

### 3.5. Effect of the Predicted High Affinity Ligands on Colony Growth and Patulin Accumulation in In Vitro Assays

The selected seven molecules were tested in in vitro assays. They did not affect *P. expansum* colony (radial) growth (Table 2), whereas patulin accumulation largely varied in presence of the investigated high affinity ligands (Table 2). Notably, saccharin and gallic acid seemed to stimulate patulin accumulation at the tested concentrations (Table 2). Conversely, meticrane, *p*-anisic acid, 6-hydroxycoumarin, and tyrosol appeared to reduce patulin accumulation. More in details, 6-hydroxycoumarin, was the most efficient dose-dependent inhibitor of patulin production (17–81%, at 12 d post inoculation (dpi), Table 2). Tyrosol behaved similarly to 6-hydroxycoumarin, being able to reduce patulin accumulation by 3–47% in a dose dependent way at 12 dpi at the tested concentrations (Table 2). Meticrane, already at the lowest tested concentration, was an efficient inhibitor of patulin production, able to reduce patulin accumulation of about 44% at all the tested concentrations (Table 2). Notably, meticrane inhibition of patulin production was independent from the dose at the tested concentrations. The solving buffer proved to not influence both fungal growth and patulin production.

### 3.6. The Effect of the Predicted High Affinity Ligands in In Vivo Assays

**Figure 5:**
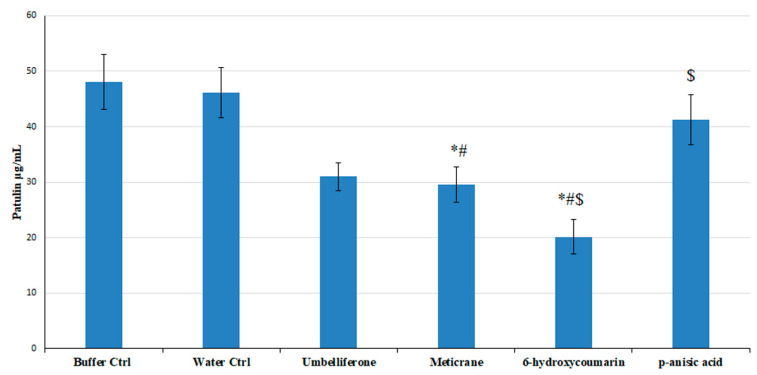
Patulin accumulation in Golden Delicious apples treated (100 µg/wound) or untreated by the selected compounds. Reported data are means ± standard errors (SE) of at least three replicates. Statistically significant differences (Turkey test, *p* ≤ 0.05) are indicated by means of symbols “*,#, $” for analyses in presence of the newly proposed ligands, namely Meticrane, 6-hydroxycoumarin and *p*-anisic acid with respect to Buffer Ctrl (consisting of PBS + NaOH), Water Ctrl (consisting of H_2_O), and the known inhibitor umbelliferone. The symbols “*,#, $” indicate a *p* ≤ 0.05 for each indicated treatment compared to Buffer control (*), Water control (#), or Umbelliferone treatment ($), respectively.

Meticrane, 6-hydroxycoumarin, tyrosol, and *p*-anisic acid, revealed to be the most efficient patulin production inhibitors in in vitro assays among the screened ligands, were tested in in vivo assays on Golden Delicious apples against disease incidence and severity (Table 3; Figure S1) and patulin accumulation (Figure 5). Tyrosol increased disease severity of about 50%, as compared to the control (data not shown) and thus, it was excluded from the following analyses. Notably, the addition of *p*-anisic acid determined a reduction of 71–28% at 8 dpi of the disease severity (lesion diameter) and 68–22% of disease incidence (infected wounds) on treated apples with respect to buffer control apples, but did not affect patulin accumulation. The addition of meticrane and 6-hydroxycoumarin did not result in a significant variation in disease incidence (Table 3, Figure S1). However, meticrane and 6-hydroxycoumarin reduced disease severity by up to 27 and 23%, respectively. Furthermore, the addition of meticrane and 6-hydroxycoumarin resulted in the reduction of patulin accumulation of about 41 and 60%, respectively (Figure 5). The solving buffer proved to not influence both disease incidence and severity and patulin accumulation.

## 4. Discussion

Reducing the presence of mycotoxins on harvested products represents an important challenge considering the need to monitor mycotoxins and chemical fungicides in fruits and vegetables, according to current legislation [5,45,58,59,60,61,62,63,64]. Patulin is a mycotoxin produced by several *Penicillium* species but mainly associated to *P. expansum* [5,45]. This mycotoxin is known for being involved in the onset of neurotoxicity, cytotoxicity, genotoxicity and gastrointestinal disorders [5,45,46,63,65]. The biosynthesis of patulin has been largely investigated [46,66], although the enzymes involved in the patulin biosynthetic pathway have not been characterized from a biochemical and structural point of view, yet. In order to find out patulin production inhibitors, we decided to structurally analyze the *P. expansum* GMC oxidoreductase, encoded by the gene *PatE*, known for being involved in the last step of patulin biosynthesis [6]. Given the fact that no crystallized structure of the investigated GMC oxidoreductase was available on the protein data bank, we performed a multi-step computational analysis that allowed us to build a 3D structural comparative model of the investigated *P. expansum* GMC oxidoreductase. By comparative analysis of the phylogenetically closest homologous GMC oxidoreductase proteins [3], it was possible to provide new clues about amino acids involved in the binding of the FAD cofactor and/or in the binding of substrates of GMC oxidoreductase superfamily members. From the sequence comparative analysis, it was possible to identify 10 blocks of conserved residues reflecting a putative role in substrate or cofactor binding and mechanism of function.

From the performed structural comparative analysis it was possible to identify a similarly located catalytic region involved in the binding of substrates and inhibitors of the sampled crystallized structures used for modelling the 3D structure of *P. expansum* GMC oxidoreductase. A pair of histidine residues (H564 and H608, *P. expansum* GMC oxidoreductase residues numbering) and two aromatic residues F563 and Y149 appeared to be crucial for the binding of patulin ligand in *P. expansum* GMC oxidoreductase. Notably, all the investigated GMC oxidoreductase homologous structures, crystallized in complex with ligands hosting aromatic functional groups, showed in their catalytic site always two histidine residues and one aromatic residue, forming an aromatic binding pocket (see i.e., (*R*)-mandelonitrile lyase in complex with benzaldehyde, “6lqy.pdb”, [32]; pyridoxine 4-oxidase in complex with 4-(aminomethyl)-5-(hydroxymethyl)-2-methylpyridin-3-ol, “4ha6.pdb”, [30]; aryl-alcohol oxidase in complex with *p*-anisic acid, “5oc1.pdb”, [29]; (*R*)-oxynitrile lyase isoenzyme 1 in complex with benzaldehyde, “3gdn.pdb”, [31]). Conversely, the fatty acid photodecarboxylase crystallized in complex with palmitate, “5ncc.pdb” [28] in correspondence of *P. expansum* GMC oxidoreductase H564, H608, F563 and Y149 hosted hydrophobic/hydrophilic (not aromatic) residues, i.e. an alanine (A576), a glutamine (Q620) an asparagine (N575) and a further alanine (A171). The cited residues, due to the lower steric hindrance (due to the lack of aromatic moieties) and to the presence of hydrophobic/hydrophilic side chains are suitable for interacting with palmitate ligand.

Notably, histidine residues and aromatic residues crucial for substrate binding located in the 3rd, 8th, and 10th blocks of conserved amino acids. As observed for the above cited crystallized structures, a variation in residues composition of 3rd, 8th and 10th amino acid blocks might reflect a change in substrate specificity; while adjacent blocks of residues remained highly conserved (see i.e., residues of blocks 1, 5 and 9, also within “5ncc.pdb” protein, crystallized in complex with palmitate).

It should be noticed that residues involved in the binding of FAD cofactor located mainly in the 1st, 3rd, 8th and 10th blocks of conserved amino acids. The 1st and 3rd amino acid blocks were enriched in glycine residues, crucial for protein function, as observed for other FAD dependent enzymes detected in all living organisms [10,67]. The 1st and 3rd amino acid blocks hosted most of residues forming the Rossmann fold (βαβαβ) [2] or βαβ mononucleotide-binding motif described as two characterizing motifs of the GMC oxidoreductase superfamily members [3]. 

The highlighted blocks of conserved amino acids extended what was previously proposed for GMC oxidoreductase superfamily in terms of mechanism and substrate specificity [3] and allowed to define a dedicated protein subgroup in which it was possible to include *P. expansum* GMC oxidoreductase phylogenetically related proteins.

Thanks to this structural comparative analysis it was possible to set up a gridbox containing all residues proposed to be involved in the binding of patulin (as well as its precursor (*E*)-ascladiol). Furthermore, it was performed a virtual screening of a chemical library that allowed to identify new high affinity ligands for *P. expansum* GMC oxidoreductase. Eight commercially available ligands out of the 76 predicted highly affinity ligands were tested in in vitro/in vivo assays. Among the investigated molecules, umbelliferone, already known for being involved in the reduction of patulin production [45], was tested for comparative purposes. 

Although none of the tested compounds reduced fungal growth, they were effective on patulin production. Particularly, *p*-anisic acid (0–33% of inhibition in the 10 µM–1 mM concentration range), meticrane (43% in the 10 µM–1 mM range) and 6-hydroxycoumarin (17–80% in the 10 µM–1 mM range), structurally related to patulin (and its precursor (*E*)-ascladiol) or to patulin production inhibitors (i.e., umbelliferone, [45]) showed to inhibit patulin production in in vitro assays, similarly to what observed for umbelliferone (22–48% in the 10 µM–1 mM concentration range). Notably, while 6-hydroxycoumarine was able to inhibit patulin production in a dose dependent way (17–80%), meticrane (an approved diuretic drug) ability in inhibiting patulin production was independent on the tested dose, showing a 43% of patulin production inhibition already at 10 µM. 

When those compounds were tested in in vivo assays, 100 µg/wound of 6-hydroxycoumarin and meticrane reduced patulin accumulation by 60 and 41 %, respectively, and disease severity up to 27% and 23%, respectively. On the contrary, *p*-anisic acid proved to be efficient against the disease, but not very efficient in reducing patulin production. This finding might be a consequence of a dilution of the compounds in apple watery tissues, since its effect on patulin biosynthesis proved to be dose-dependent. Furthermore, since *p*-anisic acid seemed to not have a direct effect on the fungus, its effect on the disease might be ascribed to an induction of host resistance as already observed for umbelliferone and quercetin [4,45] and for the structurally related salicylic acid [68,69]. 

Since the observed *p*-anisic acid effect in controlling the disease is interesting, an integrated application strategy with meticrane and/or 6-hydroxycoumarin for controlling *P. expansum* spreading and patulin accumulation in fruit tissue might be attempted and eventually confirmed by large-scale trials. Furthermore, it should be considered the possibility to use the investigated molecules, in place of other more toxic compounds, in post-harvest antifungal treatments based on dipping or drenching methods.

Notably, umbelliferone and 6-hydroxycoumarin are regioisomers and displayed significantly different activities with the latter being roughly twice as potent as the former in reducing patulin accumulation. Both regioisomers displayed concentration-dependent activities, thus confirming that the observed reduction in patulin accumulation stems from specific interactions of both hydroxycoumarins with the same protein target. Under the hypothesis that hydroxycoumarins decrease patulin production by directly inhibiting the last step of patulin biosynthesis, it might be speculated that the inhibitors displaying the higher similarity with patulin (or its precursor) should perform as the most potent inhibitors. 

**Figure 6:**
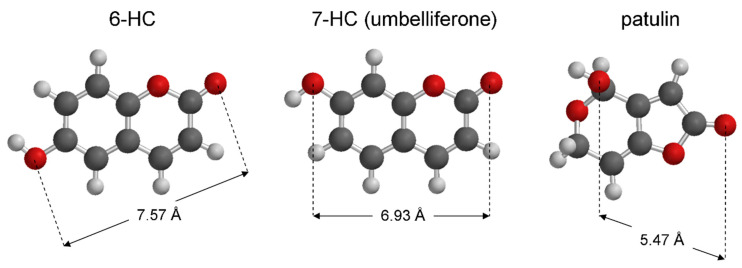
Structures of 6-hydroxycoumarin (6-HC), umbelliferone (7-hydroxycoumarin, 7-HC), and patulin represented as the corresponding most stable conformers (DFT B3LYP/6-31G*//DFT B3LYP/6-31G*); the distances between the OH and the C=O groups of each compound are highlighted.

When considering the results obtained with 6-hydroxycoumarin and 7-hydroxycoumarin (umbelliferone), the difference in potency should reflect the different orientation of the phenolic groups with the distance between the OH and carbonyl (C=O) oxygens being 7.57 Å and 6.93 Å, respectively (Figure 6). 

The two groups (OH and C=O) are closer in the global minimum patulin conformer (DFT B3LYP/6-31G*//DFT B3LYP/6-31G*; 5.47 Å) than in both inhibitors, with 6-hydroxycoumarin presenting the higher difference. Thus, this geometry parameter seems to be directly related to the observed inhibiting activities of 6-hydroxycoumarin and 7-hydroxycoumarin contrasting the above similarity principle hypothesis. On the other hand, the different orientation of the OH groups may condition the physicochemical profile of hydroxycoumarins.

While 6-hydroxycoumarin and 7-hydroxycoumarin are expected to display similar intrinsic hydrophilic/lipophilic balance (cLog*P* = 1.61 for both regioisomers; Spartan’16, Wavefunction Inc., Irvine, CA, USA), they differ in acidity [70], with the 7-hydroxycoumarin OH group being dissociated for about 50% at neutral pH conditions. Since patulin is a neutral compound (p*K*_a_ estimated to be 11.7 [71]) and does not dissociate in water, it may be hypothesized that less acidic coumarins should be more similar to patulin in this respect and could display higher inhibiting properties on its accumulation. This might explain why the less acidic coumarin 6-hydroxycoumarin is more potent than 7-hydroxycoumarin (umbelliferone).

The above considerations are highly speculative and further relevant hydroxycoumarins should be studied to highlight in a deeper detail structure-activity-relationships (SAR). However, umbelliferone and 6-hydroxycoumarin are relatively efficient agents [72] and numerous hydroxycoumarins are commercially available. The orientation of the OH group and physicochemical parameters appear as structural features possibly related to the observed inhibiting activity of hydroxycoumarins. SAR guided decoration of this promising scaffold might afford new agents for inhibiting patulin production.

## 5. Conclusions

We proposed a combined in silico/in vitro/in vivo strategy for the identification of small molecules with high affinity for *P. expansum* GMC oxidoreductase involved in the biosynthesis of the mycotoxin patulin, a common contaminant of fruit and vegetable-based products, toxic for human and animal health. Based on the described structural comparative analysis, on the features of ligands already known for their interactions with GMC oxidoreductase homologous proteins, and on the performed docking-based virtual screening of our chemical library, we identified a group of small molecules with high affinity for *P. expansum* GMC oxidoreductase. Two small molecules, among those analysed, namely 6-hydroxycoumarin (structurally related to umbelliferon, an already characterized patulin synthase inhibitor) and meticrane (an already approved drug, ranked among the best hits of our virtual screening) considerably reduced patulin accumulation in our in vitro/in vivo assays, providing a demonstration of the efficiency of the proposed approach. The presented strategy might become in the next future a reference strategy for the rapid identification of small molecules with high affinity for proteins involved also in the production of other mycotoxins. Selecting the small molecules to be screened among libraries of approved drugs/phytochemicals, in the context of chemical/drug-repurposing, will help in the rapid/effective identification of safe small molecules to be used in agriculture, for limiting fungal spread and/or mycotoxin production, without the need for other toxicity testing.

## Figures and Tables

**Table 1:** List of the seven ligands selected for evaluating in in vitro/in vivo assays their ability in reducing patulin (also reported in the table) production. Ligands are sorted by binding energy. “Binding energy” stays for the energy of the lowest energy conformation in the largest cluster identified among autodock runs. 2D structures of the assayed ligands are also reported.

Rank	Chemical Code	Structure	Name/IUPAC	Binding Energy (kcal/mol)	ChEMBL/ChEBI/Zink	Medical Indication
16	D01605	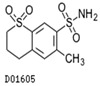	Meticrane	−8.5	https://www.ebi.ac.uk/chembl/compound_report_card/CHEMBL1318341/	Cardiovascular agent
1454	C12284	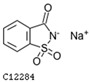	Saccharin sodium anhydrous	−6.99	https://www.ebi.ac.uk/chembl/compound_report_card/CHEMBL2219743/	/
3369	ZINC175734	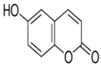	6-hydroxycoumarin	−5.88	https://zinc.docking.org/substances/ZINC000001757340/	/
4394	C02519	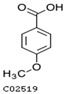	*p*-anisic acid	−5.34	https://www.ebi.ac.uk/chembl/compound_report_card/CHEMBL21932/	/
4702	C16748	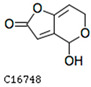	Patulin	−5.18	https://www.ebi.ac.uk/chembl/compound_report_card/CHEMBL294018/	/
5003	C10788	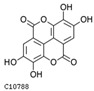	Ellagic acid	−4.99	https://www.ebi.ac.uk/chembl/compound_report_card/CHEMBL6246/	Antiproliferative and antioxidant agent
5686	C06044	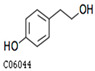	4-Hydroxyphenylethanol (tyrosol)	−4.58	https://www.ebi.ac.uk/chembl/compound_report_card/CHEMBL53566/	/
6154	C01424	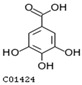	Gallic acid	−4.29	https://www.ebi.ac.uk/chembl/compound_report_card/CHEMBL288114/	/

**Table 2:** In vitro colony growth (estimated as radial growth, mm) and patulin production (μg/mL) after 12 days post inoculation (dpi). Mean values and standard errors (SE) of at least three replicates are reported. Superscript letters (a–i) in each cell indicate whether the comparison (Tuckey test) between the corresponding pair of samples in a certain column returned a statistically significant difference (*p* ≤ 0.05, different letters) or not (same letter).

Colony Growth (mm) ± SE				Patulin Accumulation ± SE (μg/mL)
	4 dpi	8 dpi	12 dpi	12 dpi
Water ctrl (H_2_O)	23 ± 3 ^a^	54 ± 4 ^a^	78 ± 5 ^a^	1130 ± 40 ^d^
Buffer ctrl (PBS + NaOH)	25 ± 1 ^a^	55 ± 1.5 ^a^	80 ± 3 ^a^	1190 ± 70 ^d^
Umbelliferone 0.01 mM	24.5 ± 0.5 ^a^	54.8 ± 0.3 ^a^	79.3 ± 2.0 ^a^	890 ± 28 ^e^
Umbelliferone 0.1 mM	24.3 ± 0.3 ^a^	54.7 ± 1.0 ^a^	79.5 ± 2.1 ^a^	810 ± 30 ^e^
Umbelliferone 0.5 mM	24.6 ± 0.8 ^a^	54.6 ± 0.8 ^a^	79.8 ± 1.9 ^a^	743 ± 40 ^h^
Umbelliferone 1 mM	24.6 ± 0.7 ^a^	54.8 ± 1.2 ^a^	79.5 ± 2.2 ^a^	590 ± 60 ^g^
Meticrane 0.01 mM	22.5 ± 3 ^a^	53 ± 4 ^a^	79 ± 6 ^a^	650 ± 30 ^g^
Meticrane 0.1 mM	23 ± 4 ^a^	53 ± 5 ^a^	78 ± 5 ^a^	612.3 ± 26 ^g^
Meticrane 0.5 mM	23 ± 3 ^a^	53 ± 4 ^a^	78 ± 6 ^a^	640 ± 30 ^g^
Meticrane 1 mM	23 ± 4 ^a^	54 ± 4 ^a^	78 ± 6 ^a^	610 ± 40 ^g^
Anisic acid 0.01 mM	23 ± 3 ^a^	53 ± 4 ^a^	79 ± 3 ^a^	1100 ± 120 ^d^
Anisic acid 0.1 mM	23 ± 3 ^a^	53 ± 4 ^a^	79 ± 4 ^a^	910 ± 80 ^e^
Anisic acid 0.5 mM	24 ± 4 ^a^	54 ± 4 ^a^	77 ± 5 ^a^	840 ± 110 ^e^
Anisic acid 1 mM	22 ± 4 ^a^	54 ± 4 ^a^	78 ± 5 ^a^	760 ± 90 ^f^
Saccharin 0.01 mM	23 ± 4 ^a^	56 ± 3 ^a^	80 ± 3 ^a^	1180 ± 50 ^d^
Saccharin 0.1 mM	24 ± 4 ^a^	56 ± 3 ^a^	80 ± 4 ^a^	1310 ± 70 ^c^
Saccharin 0.5 mM	24.5 ± 2.0 ^a^	56 ± 4 ^a^	80 ± 5 ^a^	1510 ± 80 ^a^
Saccharin 1 mM	24 ± 3 ^a^	56 ± 3 ^a^	79 ± 3 ^a^	1420 ± 30 ^b^
6-hydroxycoumarin 0.01 mM	21 ± 3 ^a^	53 ± 3 ^a^	78 ± 4	834 ± 70 ^e^
6-hydroxycoumarin 0.1 mM	22 ± 3 ^a^	54 ± 4 ^a^	79 ± 4 ^a^	710 ± 50 ^f^
6-hydroxycoumarin 0.5 mM	22.3 ± 2.0 ^a^	52 ± 4 ^a^	76 ± 3 ^a^	346 ± 25 ^h^
6-hydroxycoumarin 1 mM	22 ± 3 ^a^	52 ± 3 ^a^	77 ± 4 ^a^	240 ± 30 ^i^
Tyrosol 0.01 mM	24 ± 3 ^a^	55 ± 4 ^a^	79 ± 5 ^a^	1120 ± 60 ^d^
Tyrosol 0.1 mM	24 ± 3 ^a^	56 ± 4 ^a^	79 ± 4 ^a^	950 ± 50 ^e^
Tyrosol 0.5 mM	24 ± 3 ^a^	55 ± 4 ^a^	78 ± 4 ^a^	690 ± 30 ^f^
Tyrosol 1 mM	24 ± 3 ^a^	56 ± 4 ^a^	78 ± 6 ^a^	600 ± 40 ^g^
Gallic acid 0.01 mM	21 ± 4 ^a^	54 ± 5 ^a^	77 ± 6 ^a^	900 ± 50 ^e^
Gallic acid 0.1 mM	22 ± 2 ^a^	55 ± 3 ^a^	81 ± 5 ^a^	1020 ± 70 ^d^
Gallic acid 0.5 mM	24 ± 4 ^a^	60 ± 5 ^a^	81 ± 5 ^a^	1390 ± 50 ^b^
Gallic acid 1 mM	24 ± 3 ^a^	59 ± 5 ^a^	79 ± 4 ^a^	1473 ±22 ^a^
Ellagic acid 0.01 mM	23.8 ± 2.7 ^a^	59.5 ± 4 ^a^	81 ± 5 ^a^	1190 ± 80 ^d^
Ellagic acid 0.1 mM	24 ± 3 ^a^	59.7 ± 2.5 ^a^	81.4 ± 2.4 ^a^	1320 ± 40 ^b^
Ellagic acid 0.5 mM	23.7 ± 1.9 ^a^	60 ± 3 ^a^	82 ± 3 ^a^	1280 ± 60 ^b^
Ellagic acid 1 mM	24 ± 3 ^a^	60 ± 3 ^a^	82 ± 4 ^a^	1300 ± 30 ^b^

**Table 3:** Disease incidence (expressed as % of the infected wounds) and severity (expressed as lesion diameter, reported in mm) on Golden Delicious apples inoculated by *P. expansum*. Mean values and standard errors (SE) of at least three replicates are reported. Superscript letters (a–c) in each cell indicate whether the comparison (Duncan’s Multiple Range Test, DMRT test) between the corresponding pair of samples in a certain column returned a statistically significant difference (*p* ≤ 0.05, different letters) or not (same letter).

	2 dpi		4 dpi		8 dpi	
	Infected Wounds ± SE (%)	Lesion Diameters ± SE (mm)	Infected Wounds ± SE (%)	Lesion Diameters ± SE (mm)	Infected Wounds ± SE (%)	Lesion Diameters ± SE (mm)
Water ctrl (H_2_O)	17 ± 3 ^a^	11 ± 1 ^a^	82 ± 2 ^a^	55 ± 2 ^a^	100 ^a^	105 ± 4 ^a^
Buffer ctrl (PBS+NaOH)	16 ± 3 ^a^	12.2 ± 1.4 ^a^	80 ± 3 ^a^	57 ± 3 ^a^	100 ^a^	112 ± 5 ^a^
Umbelliferone	9 ± 3 ^b^	9.1 ± 1.2 ^a^	75 ± 6 ^a^	53 ± 4 ^a^	100 ^a^	107 ± 4 ^a^
Meticrane	17 ± 2 ^a^	8 ± 1.5 ^b^	80.5 ± 2 ^a^	47 ± 2 ^b^	100 ^a^	83 ± 2 ^b^
*p*-anisic acid	5 ± 3 ^b^	3.5 ± 0.5 ^c^	30 ± 3 ^b^	42 ± 1 ^c^	78 ± 1 ^b^	76 ± 3 ^c^
6-hydroxycoumarin	17 ± 1 ^a^	10 ± 0.5 ^a^	83 ± 3 ^a^	46 ± 3 ^b^	100 ^a^	86 ± 4 ^b^

## Supplementary Materials

The following are available online at https://www.mdpi.com/2079-7737/10/1/21/s1, Figure S1: In vitro/in vivo viability/infection assays, Table S1: List of residues within 4 Å from the crystallized or docked ligands within the indicated homologous crystallized structures sampled by using pGenThreader and I-tasser, for comparative modelling purposes. In the last column, residues of the 3D comparative model of the investigated *P. expansum* GMC oxidoreductase (PatE, patulin synthase) within 4 Å from the docked patulin are reported, Table S2: List of 10 small molecules chosen among the first 20 best hits based on the lowest calculated binding energy, on the presence of aromatic moieties and on their similarity with patulin and (*E*)-ascladiol, Table S3: List of 11 small molecules energetically/structurally related to the saccharin molecule, which is among the ligands more similar to patulin and (*E*)-ascladiol, within the screened library, Table S4: List of 11 small molecules structurally/energetically related to the umbelliferone molecule, able to control patulin production, Table S5: List of 11 small molecules structurally/energetically related to the *p*-anisic acid, known for being able to inhibit 5oc1.pdb used as a protein template for comparative modelling of *P. expansum* GMC oxidoreductase, Table S6: List of 11 small molecules structurally/energetically related to the ellagic acid, structurally related to the umbelliferone molecule, able to control patulin production, Table S7: List of 11 small molecules structurally/energetically related to tyrosol, similar in structure to p-anisic acid (able to inhibit 5oc1.pdb) and benzaldehyde (substrate for 3gdn.pdb and 6lqy.pdb), which are among the best protein templates to be used for the comparative modelling of *P. expansum* GMC oxidoreductase, Table S8: List of 11 small molecules structurally/energetically related to the gallic acid, similar in structure to the *p*-anisic acid (able to inhibit 5oc1.pdb) and benzaldehyde (substrate for 3gdn.pdb and 6lqy.pdb), which are among the best protein templates to be used for the comparative modelling of *P. expansum* GMC oxidoreductase.
