# Supplementary material for: Targeting Penicillium expansum GMC Oxidoreductase with High Affinity Small Molecules for Reducing Patulin Production
Source: Biology (Basel). 2020 Dec 31;10(1):21. doi: 10.3390/biology10010021 (PMC7824139; doi:10.3390/biology10010021)
Supplement: Supplementary file 1 [file biology-10-00021-s001.pdf]

Supp. Figure S1: *In vitro/in vivo* viability/infection assays. Colony growth after 12 days post-inoculation (dpi) of *P. expansum* on PDA medium amended with buffer solution (positive control, A), water (negative control, B), 1 mM meticrane (C), 1 mM 6-hydroxycoumarine (D), 1 mM *p*-anisic acid (E). *P. expansum* lesion diameters on positive (buffer, F) and negative (water, G) control apples at 8 dpi. Lesion diameters at 8 dpi on apples treated by meticrane, 6-hydroxycoumarin and *p*-anisic acid (100 µg/wound for all the three compounds) (H-J).

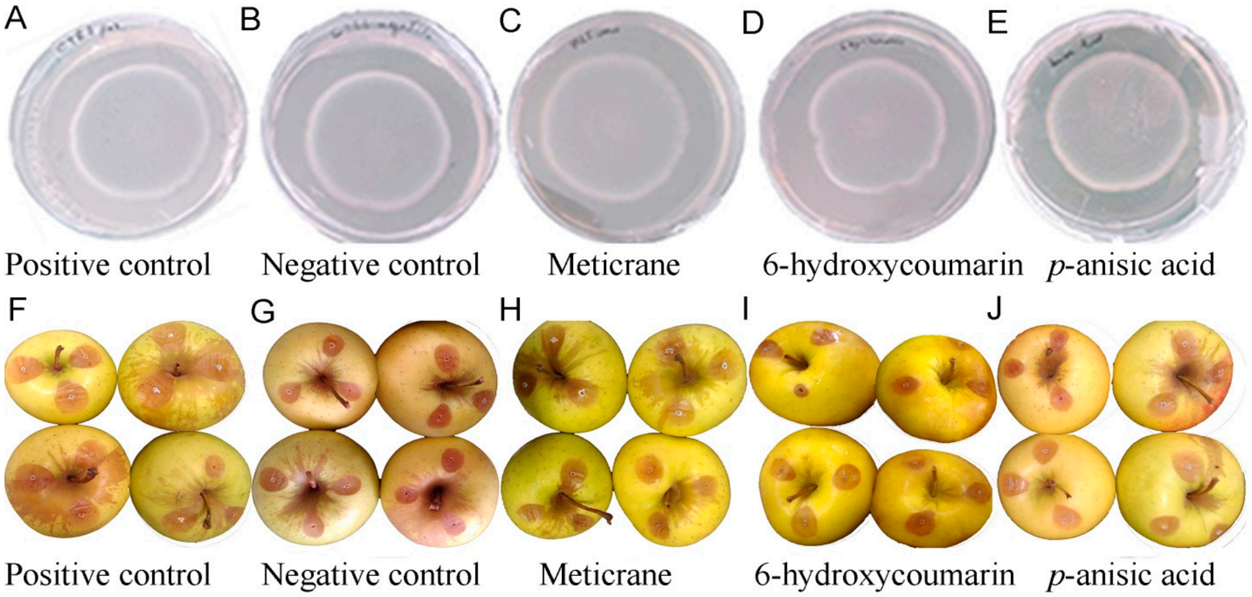

Supp. Table S1: List of residues within 4 Å from the crystallized or docked ligands within the indicated homologous crystallized structures sampled by using pGenThreader and I-tasser, for comparative modelling purposes. In the last column, residues of the 3D comparative model of the investigated *P. expansum* GMC oxidoreductase (PatE, patulin synthase) within 4 Å from the docked patulin are reported.

Protein	*Pleurotus eryngii*, aryl-alcohol oxidase (5oc1.pdb)	*Mesorhizobium loti*, pyridoxine 4-oxidase (4ha6.pdb)	*Chlorella variabilis*, fatty acid photodecarboxylase (5ncc.pdb)	*Prunus dulcis*, endo-deglycosylated hydroxynitrile lyase isozyme 5 (6lqy.pdb)	*Prunus dulcis*, hydroxynitrile lyase (3gdn.pdb)	*P. expansum*, GMC oxidoreductase (comparative 3D model)
Crystallized ligand	4-methoxybenzoic acid (ANN)	pyridoxamine (PXM)	palmitate (PLM)	benzaldehyde (HBX)	benzaldehyde (HBX)	patulin
Residues						Gln100
Residues						Tyr104
Residues		Trp64	Ile130			
Residues						Ans148
Residues	Tyr92	Ala105	Ala171	Ala111	Ala111	Tyr149
Residues						Ala151
Residues				Arg301		
Residues			Leu386			
Residues					Val316	
Residues		Ser330				
Residues	Ile357					
Residues				Val329	Cys328	
Residues				Leu331	Phe 330	
Residues		Arg451				
Residues		Val453				
Residues		Gly455				
Residues		Met456				
Residues		Ala457				
Residues					Phe342	
Residues		Gly462				
Residues	Ile391					Tyr453
Residues			Thr465			
Residues	Ser393		Tyr466			
Residues			Phe469			
Residues	Phe397					
Residues			Thr484	His358	His357	
Residues	Thr413		Gln486			
Residues	Ile500	His460		Tyr458	Tyr457	Ile562
Residues	Phe501	His461	Ans575	Trp459	Trp458	Phe563
Residues	His502	His462				His564
Residues	His546	Pro504		His498	His497	His608

Supp. Table S2: List of 10 small molecules chosen among the first 20 best hits based on the lowest calculated binding energy, on the presence of aromatic moieties and on their similarity with patulin and (*E*)-ascladiol.

Rank	Chemical code	Structure	Name	Binding energy (kcal/mol)	ChEMBL	Indication
1	D01896	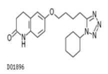	Cilostazol	-9.47	https://www.ebi.ac.uk/chembl/compound_report_card/CHEMBL799/	Cardiovascular agent
2	D00848	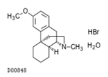	Dextromethorphan hydrobromide	-9.19	https://www.ebi.ac.uk/chembl/compound_report_card/CHEMBL1256818/	Neuropsychiatric agent
3	D01506	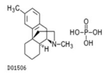	Dimemorfan phosphate	-9.01	https://www.ebi.ac.uk/chembl/compound_report_card/CHEMBL2106325/	Antitussive
7	D03648	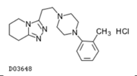	Dapoxetine hydrochloride	-8.74	https://www.ebi.ac.uk/chembl/g/#search_results/all/query=Dapoxetine%20hydrochloride	Neuropsychiatric agent
8	D00381	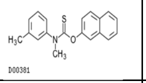	Tolnaftate	-8.73	https://www.ebi.ac.uk/chembl/compound_report_card/CHEMBL83668/	Antifungal agent
12	D00653	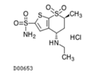	Dorzolamide hydrochloride	-8.6	https://www.ebi.ac.uk/chembl/compound_report_card/CHEMBL1201162/	Cardiovascular agent
13	D01876	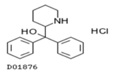	Pipradrol hydrochloride	-8.6	https://www.ebi.ac.uk/chembl/compound_report_card/CHEMBL2106720/	Neuropsychiatric agent
16	D01605	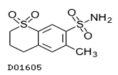	Meticrane	-8.5	https://www.ebi.ac.uk/chembl/compound_report_card/CHEMBL1318341/	Cardiovascular agent
17	D01056	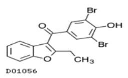	Benzbromarone	-8.48	https://www.ebi.ac.uk/chembl/compound_report_card/CHEMBL388590/	Gout suppressant
18	D01550	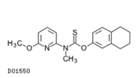	Ciranaftate	-8.39	https://www.ebi.ac.uk/chembl/compound_report_card/CHEMBL1591365/	Antifunga agent

Supp. Table S3: List of 11 small molecules energetically/structurally related to the saccharin molecule, which is among the ligands more similar to patulin and (*E*)-ascladiol, within the screened library.

Rank	Chemical code	Structure	Name	Binding energy (kcal/mol)	ChEMBL/ChEBI	Indication
1449	C10094	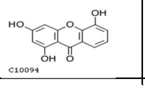	1,3,5-Trihydroxyxanthone	-6.99	https://www.ebi.ac.uk/chembl/compound_report_card/CHEMBL365234/	/
1450	C10272	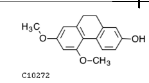	Orchinol	-6.99	https://www.ebi.ac.uk/chebi/searchId.do?chebiId=CHEBI:7779	/
1451	C10300	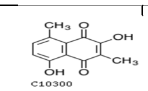	Aristolindiquinone	-6.99	https://www.ebi.ac.uk/chebi/searchId.do?chebiId=CHEBI:2823	/
1452	C11018	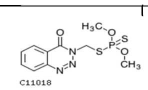	Azinphos-methyl	-6.99	https://www.ebi.ac.uk/chembl/compound_report_card/CHEMBL530115/	/
1453	C11432	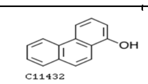	1-Hydroxyphenanthrene	-6.99	https://www.ebi.ac.uk/chebi/searchId.do?chebiId=CHEBI:27528	/
1454	C12284	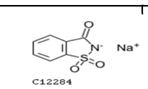	Saccharin sodium anhydrous	-6.99	https://www.ebi.ac.uk/chembl/compound_report_card/CHEMBL2219743/	/
1455	C14137	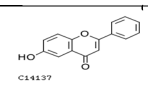	6-Hydroxyflavone	-6.99	https://www.ebi.ac.uk/chembl/compound_report_card/CHEMBL138649/	/
1456	C14905	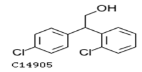	2-Chloro-beta-(4-chlorophenyl)phenethyl alcohol	-6.99	https://www.ebi.ac.uk/chembl/compound_report_card/CHEMBL138649/	/
1457	C15210	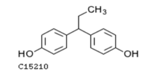	1,1-Bis(4-hydroxyphenyl)propane	-6.99	https://www.ebi.ac.uk/chebi/searchId.do?chebiId=CHEBI:79723	/
1458	D00602	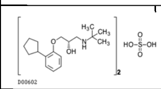	Penbutolol sulfate	-6.99	https://www.ebi.ac.uk/chembl/compound_report_card/CHEMBL2361370/	Cardiovascular agent
1459	D00773	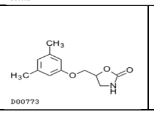	Metaxalone	-6.99	https://www.ebi.ac.uk/chembl/compound_report_card/CHEMBL1079604/	Muscle relaxant

Supp. Table S4: List of 11 small molecules structurally/energetically related to the umbelliferone molecule, able to control patulin production.

Rank	Chemical code	Structure	Name/ IUPAC	Binding energy (kcal/mol)	ChEMBL	Indication
3147	C09315	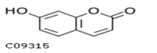	Umbelliferone	-6	https://www.ebi.ac.uk/chembl/compound_report_card/CHEMBL51628/	/
3364	D03799	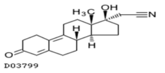	Dienogest	-5.89	https://www.ebi.ac.uk/chembl/compound_report_card/CHEMBL1201864/	/
3366	C03431	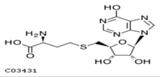	S-Inosyl-L-homocysteine	-5.89	https://www.ebi.ac.uk/chembldb/compound/inspect/CHEMBL559715	/
3367	C05648	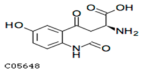	5-Hydroxy-N-formylkynurenine	-5.88	https://www.ebi.ac.uk/chebi/searchId.do?chebiId=CHEBI:2065	/
3368	C05851	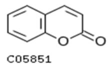	Coumarin	-5.88	https://www.ebi.ac.uk/chembl/compound_report_card/CHEMBL6466/	/
3369	ZINC175734	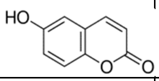	6-Hydroxycoumarin	-5.88	https://zinc.docking.org/substances/ZINC000001757340/	/
3370	C08533	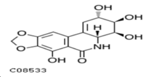	Narciclasine	-5.88	https://www.ebi.ac.uk/chembl/compound_report_card/CHEMBL98745/	/
3371	C09863	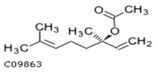	Linalyl acetate	-5.88	https://www.ebi.ac.uk/chebi/searchId.do?chebiId=CHEBI:6469	/
3372	C10938	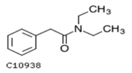	N,N-Diethylphenylacetamide	-5.88	https://www.ebi.ac.uk/chembl/compound_report_card/CHEMBL1887332/	/
3373	C11403	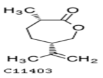	(3S,6R)-6-Isopropenyl-3-methyl-2-oxo-oxepanone	-5.88	https://www.ebi.ac.uk/chebi/searchId.do?chebiId=CHEBI:64231	/
3374	C13709	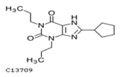	8-Cyclopentyl-1,3-dipropylxanthine	-5.88	https://www.ebi.ac.uk/chembl/compound_report_card/CHEMBL183/	/

Supp. Table S5: List of 11 small molecules structurally/energetically related to the *p*-anisic acid, known for being able to inhibit 5oc1.pdb used as a protein template for comparative modelling of *P. expansum* GMC oxidoreductase.

Rank	Chemical code	Structure	Name/IUPAC	Binding energy (kcal/mol))	ChEMBL	Indication
4389	D02109	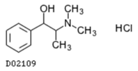	dl-Methylephedrine hydrochloride	-5.35	https://www.ebi.ac.uk/chebi/searchId.do?chebiId=CHEBI:79398	Cardiovascular agent
4390	D03908	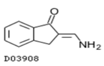	Drinidene	-5.35	https://www.ebi.ac.uk/chembl/compound_report_card/CHEMBL4297145/	Analgesic
4391	D04012	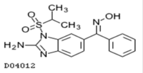	Enviroxime	-5.35	https://www.ebi.ac.uk/chembl/compound_report_card/CHEMBL283403/	Antiviral
4392	C01173	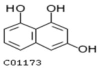	1,3,8-Trihydroxynaphthalene	-5.34	https://www.ebi.ac.uk/chebi/searchId.do?chebiId=CHEBI:18393	/
4393	C01249	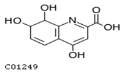	7,8-Dihydro-7,8-dihydroxykynurenate	-5.34	https://www.ebi.ac.uk/chebi/searchId.do?chebiId=CHEBI:17109	/
4394	C02519	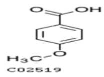	*p*-anisic acid	-5.34	https://www.ebi.ac.uk/chembl/compound_report_card/CHEMBL21932/	/
4395	C03493	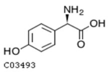	D-4-Hydroxyphenylglycine	-5.34	https://www.ebi.ac.uk/chebi/searchId.do?chebiId=CHEBI:15695	/
4396	C05512	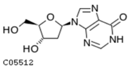	Deoxyinosine	-5.34	https://www.ebi.ac.uk/chembl/compound_report_card/CHEMBL1229971/	/
4397	C06304	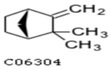	Camphene	-5.34	https://www.ebi.ac.uk/chembl/compound_report_card/CHEMBL510535/	/
4398	C07714	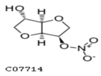	Isosorbide mononitrate	-5.34	https://www.ebi.ac.uk/chembl/compound_report_card/CHEMBL1311/	/
4399	C08027	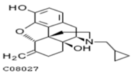	Nalmefene	-5.34	https://www.ebi.ac.uk/chembl/compound_report_card/CHEMBL982/	Oppioid receptor inhibitor

Supp. Table S6: List of 11 small molecules structurally/energetically related to the ellagic acid, structurally related to the umbelliferone molecule, able to control patulin production.

Rank	Chemical code	Structure	Name/ IUPAC	Binding energy (kcal/mol)	ChEMBL/ChEBI/Zinc	Indication
4998	C07480	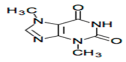 C07480	Theobromine	-4.99	https://www.ebi.ac.uk/chembl/compound_report_card/CHEMBL1114/	Stimulant of the nervous system
4999	C07770	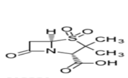 C07770	Sulbactam	-4.99	https://www.ebi.ac.uk/chembl/compound_report_card/CHEMBL403/	Antimicrobial agent
5000	C08432	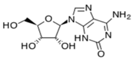 C08432	Isoguanosine	-4.99	https://www.ebi.ac.uk/chembl/compound_report_card/CHEMBL1688963/	/
5001	C10287	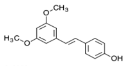 C10287	Pterostilbene	-4.99	https://www.ebi.ac.uk/chembl/compound_report_card/CHEMBL83527/	/
5002	C10496	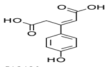 C10496	Sphagnum acid	-4.99	https://www.ebi.ac.uk/chebi/searchId.do?chebiId=CHEBI:9220	/
5003	C10788	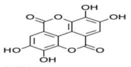 C10788	Ellagic acid	-4.99	https://www.ebi.ac.uk/chembl/compound_report_card/CHEMBL6246/	Antiproliferative and antioxidant agent
5004	C10866	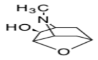 C10866	Scopoline	-4.99	https://www.ebi.ac.uk/chebi/searchId.do?chebiId=CHEBI:9059	Anticholinergic effect
5005	D01124	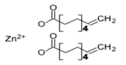 D01124	Zinc undecylenate	-4.99	https://www.ebi.ac.uk/chembl/compound_report_card/CHEMBL4303341/	Antifungal agent
5006	C13694	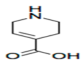 C13694	Isoguvacine	-4.99	https://www.ebi.ac.uk/chembl/compound_report_card/CHEMBL39071/	GABA A receptor agonist
5007	C14411	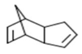 C14411	Dicyclopentadiene	-4.99	https://www.ebi.ac.uk/chembl/compound_report_card/CHEMBL1570502/	/
5008	C14839	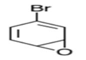 C14839	Bromobenzene-3,4-epoxide	-4.99	https://www.ebi.ac.uk/chebi/searchId.do?chebiId=CHEBI:34589	/

Supp. Table S7: List of 11 small molecules structurally/energetically related to tyrosol, similar in structure to p-anisic acid (able to inhibit 5oc1.pdb) and benzaldehyde (substrate for 3gdn.pdb and 6lqy.pdb), which are among the best protein templates to be used for the comparative modelling of *P. expansum* GMC oxidoreductase.

Rank	Chemical code	Structure	Name/ IUPAC	Binding energy (kcal/mol)	ChEMBL/ChEBI/Zinc	Indication
5681	C00881	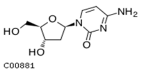	2'-Deoxycytidine	-4.58	https://www.ebi.ac.uk/chembl/compound_report_card/CHEMBL66115/	/
5682	C01892	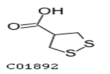	Asparagusic acid	-4.58	https://www.ebi.ac.uk/chembl/compound_report_card/CHEMBL3581910/	/
5683	C04673	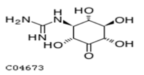	1D-1-Guanidino-1-deoxy-3-dehydro-scyllo-inositol	-4.58	https://www.ebi.ac.uk/chebi/searchId.do?chebiId=CHEBI:17845	/
5684	C04796	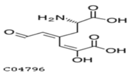	4-(L-Alanin-3-yl)-2-hydroxy-cis,cis-muconate 6-semialdehyde	-4.58	https://www.ebi.ac.uk/chebi/searchId.do?chebiId=CHEBI:16098	/
5685	C05332	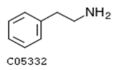	Phenylethylamine	-4.58	https://www.ebi.ac.uk/chembl/compound_report_card/CHEMBL610/	/
5686	C06044	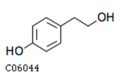	4-Hydroxyphenylethanol (tyrosol)	-4.58	https://www.ebi.ac.uk/chembl/compound_report_card/CHEMBL53566/	/
5687	C06193	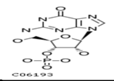	Guanosine 3'-phosphate	-4.58	https://www.ebi.ac.uk/chebi/searchId.do?chebiId=CHEBI:28072	/
5688	C07216	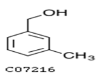	3-Methylbenzyl alcohol	-4.58	https://www.ebi.ac.uk/chebi/searchId.do?chebiId=CHEBI:27995	/
5689	C07642	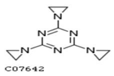	Triethylenemelamine	-4.58	https://www.ebi.ac.uk/chembl/compound_report_card/CHEMBL502384/	/
5690	C08556	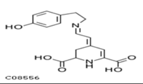	Miraxanthin-III	-4.58	https://www.ebi.ac.uk/chebi/searchId.do?chebiId=CHEBI:6947	/
5691	C08735	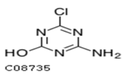	2-Chloro-4-hydroxy-6-amino-1,3,5-triazine	-4.58	https://www.ebi.ac.uk/chebi/searchId.do?chebiId=CHEBI:27797	/

Supp. Table S8: List of 11 small molecules structurally/energetically related to the gallic acid, similar in structure to the *p*-anisic acid (able to inhibit 5oc1.pdb) and benzaldehyde (substrate for 3gdn.pdb and 6lqy.pdb), which are among the best protein templates to be used for the comparative modelling of *P. expansum* GMC oxidoreductase.

Rank	Chemical code	Structure	Name/ IUPAC	Binding energy (kcal/mol)	ChEMBL/ChEBI/ Zink	Indication
6149	C11159	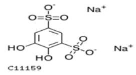	1,2-Dihydroxybenzene-3,5-disulfonic acid disodium salt (tiron)	-4.3	https://www.ebi.ac.uk/chembl/compound_report_card/CHEMBL110526/	/
6150	C11283	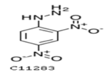	2,4-Dinitrophenylhydrazine	-4.3	https://www.ebi.ac.uk/chembl/compound_report_card/CHEMBL352799/	/
6151	D00393	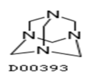	Methenamine	-4.3	https://www.ebi.ac.uk/chembl/compound_report_card/CHEMBL1201270/	Antibacterial activity
6152	D01607	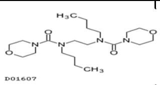	Theraptique	-4.3	https://www.ebi.ac.uk/chembl/compound_report_card/CHEMBL2104294/	Respiratory stimulant
6153	C00386	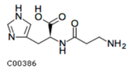	Nalpha-(beta-alanyl)-L-histidine	-4.29	https://www.ebi.ac.uk/chembl/compound_report_card/CHEMBL242948/	/
6154	C01424	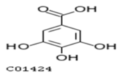	Gallic acid	-4.29	https://www.ebi.ac.uk/chembl/compound_report_card/CHEMBL288114/	/
6155	C01468	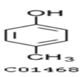	4-Hydroxytoluene	-4.29	https://www.ebi.ac.uk/chembl/compound_report_card/CHEMBL16645/	/
6156	C02965	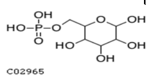	D-Hexose 6-phosphate	-4.29	https://www.ebi.ac.uk/chebi/searchId.do?chebiId=CHEBI:4195	/
6157	C03007	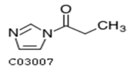	N-Propanoylimidazole	-4.29	https://www.ebi.ac.uk/chebi/searchId.do?chebiId=CHEBI:27449	/
6158	C03338	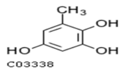	2,3,5-Trihydroxytoluene	-4.29	https://www.ebi.ac.uk/chembl/compound_report_card/CHEMBL4102748/	/
6159	C03387	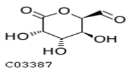	D-Glucurono-6,2-lactone	-4.29	https://www.ebi.ac.uk/chebi/searchId.do?chebiId=CHEBI:17039	/
